# Phase-Assisted Tailored Conductivity of Doped Ceria Electrolytes to Boost SOFC Performance

**DOI:** 10.1021/acsami.3c08146

**Published:** 2023-08-09

**Authors:** Muhammad S. Arshad, Caren Billing, David G. Billing, Wanbing Guan

**Affiliations:** † Molecular Sciences Institute, School of Chemistry, University of the Witwatersrand, Private Bag X3, Johannesburg 2050, South Africa; ‡ Department of Chemical Sciences, University of Johannesburg, Doornfontein, Johannesburg 2028, South Africa; § Ningbo Institute of Material Technology and Engineering, Chinese Academy of Sciences, Ningbo 315201, China

**Keywords:** fuel cell, electrolyte, codoping, phase change, conductivity, power density

## Abstract

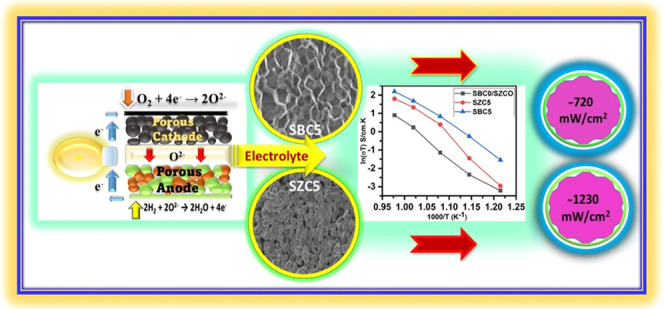

Efforts to lower the operating temperature of solid oxide fuel cells include producing electrolytes that are sufficiently conductive and stable below 600 °C. Doped ceria is one such electrolyte being considered. During this study, codoped ceria powders (Ce_0.8_Sm_0.2–*x*_M*_x_*O_2−δ_, M = Bi^3+^, Zn^2+^ and *x* = 0, 0.05, 0.1, 0.15, 0.2) were prepared via coprecipitation by the addition of sodium carbonate and annealed at 800 and 1200 °C, respectively. Poor solubility of the codopants in the ceria was observed for samples annealed at 800 °C, resulting in a mixed-phase product including stable phases of the oxides of these codopants. A second-stage partial incorporation of these codopants into the ceria lattice was observed when the annealing temperature was increased to 1200 °C, with both codopants forming cubic-type phases of their respective oxides. Materials were characterized using X-ray diffraction (XRD), Raman spectroscopy, and Fourier transform infrared spectroscopy (FTIR), as well as scanning electron microscopy (SEM) for structural and morphological investigations. The oxide ion conductivity was evaluated using electrochemical impedance spectroscopy between 550 and 750 °C. Fuel cell performance tests of selected samples (annealed at 1200 °C) showed remarkable improvement in peak power densities when the test temperature was increased from 500 to 600 °C (∼720 mW/cm^2^ for Ce_0.8_Sm_0.15_Bi_0.05_O_2−δ_ and ∼1230 mW/cm^2^ for Ce_0.8_Sm_0.15_Zn_0.05_O_2−δ_), indicating possible contribution from the distinct cubic-type oxide phases of the codopants in performance enhancement.

## 1 Introduction

Solid oxide fuel cells (SOFCs) can have a huge positive impact on the world’s changing environment due to global warming and play an important role in realizing the United Nations’ Sustainable Development Goal 7, calling to “ensure access to affordable, reliable, sustainable and modern energy for all”.^1^ The ability of SOFCs to operate directly on hydrocarbon fuels eliminates the need for reformers, making them appealing for commercial applications.^2,3^ This is possible if the bottlenecks toward their commercialization can be tackled, one of them being their high operating temperature (800–1000 °C), which itself brings associated disadvantages, such as rapid deterioration of cell components and their compatibility. A component of prime importance is the electrolyte that plays a significant role in determining the SOFC operating temperature. Traditionally used yttria-stabilized zirconia (YSZ) shows acceptable ionic conduction but only at high temperature (≥800 °C), with an unsatisfactory conductivity at low temperature (≤600 °C).^4,5^

Ceria is an important oxide ion conductor. However, it shows poor conductivity in pure form at low temperature (≤600 °C).^6^ Doped ceria has received much attention in recent decades for electrolytic applications in SOFCs due to its notable ionic conductivity in the low-temperature regime (600 °C or lower) and good compatibility with fuel electrode materials.^7^ Nanostructured ceria shows high thermal stability as well as catalytic activity and impedes carbon formation when hydrocarbon fuels are used.^8,9^ Although Ce_0.8_Sm_0.2_O_2−δ_ (SDC) has demonstrated impressive oxide ion conductivity with appreciable fuel cell performance,^10^ efforts have been made and are still in progress to introduce codopants to replace costly rare earth constituent metals and to further enhance the ion migration activity at lower temperature while improving other features like density, electrode–electrolyte compatibility, durability, and performance.^11−13^ Various examples exist where ceria doped with aliovalent cations has shown appreciable results.^11,12,14,15^ For example, Wu et al.^14^ observed notable ionic conduction for aliovalent cations (Sm^3+^, Mg^2+^, Ca^2+^, Sr^2+^, Ba^2+^) codoped ceria electrolytes. Ceria carbonate composite electrolytes have attracted considerable attention in recent years for low-temperature applications due to the possible role of carbonate in conductivity enhancement. A novel core–shell SDC/Na_2_CO_3_ composite electrolyte prepared by Wang et al.^16^ obtained a conductivity of over 0.1 S/cm at a temperature slightly above 300 °C, ascribing it to the development of an interface between carbonate and ceria phases for fast ion transport. Various mechanisms of oxide ion transport through carbonate are discussed in (17). A unique advantage of carbonate is that it allows hybrid conduction of ions (H^+^/O^2–^),^18^ and the presence of carbonate around ceria transforms it into a multi-ion [O^2–^, H^+^, CO_3_^2–^] conductive electrolyte.^19^ Apart from its beneficial role in conductivity enhancement, it acts as a sintering aid, a pore filler to increase the density of the electrolytic material, an electron insulator, an electrode/electrolyte glue, and an accelerator for the oxygen reduction reaction.^20^

Bi_2_O_3_ received a lot of attention for SOFC electrolytic applications due to its exceptionally high oxide ion conductivity, but due to certain serious drawbacks, interest has declined.^21^ Some studies attempted to dope Bi^3+^ into the ceria lattice or introduce Bi^3+^ into ceria electrolytes to make use of the high oxide conducting ability of Bi_2_O_3_.^22,23^ For example, Zhao et al.^22^ observed a notable increase in oxide transport by incorporating a small amount of Bi^3+^ into SDC (conductivity of Ce_0.8_Sm_0.1_Bi_0.1_O_1.9_ ≈ 3.98 × 10^–2^ S/cm at 750 °C) compared to that of SDC (conductivity of Ce_0.8_Sm_0.2_O_1.9_ ≈ 2.2 × 10^–2^ S/cm at 750 °C). However, Bi^3+^ solubility in the ceria lattice, and hence the formation of a single-phase Bi^3+^-doped ceria system, is dependent on temperature and the synthetic methodology. As noted by Accardo et al.,^23^ limited solubility of Bi^3+^ in the ceria lattice is expected because of the general violation of the Hume–Rothery rules for solid solutions, also applicable to ceramic systems. Moreover, the gradual evolution of Bi^3+^-related phases was observed for CeO_2_–Bi_2_O_3_ systems over time or upon high-temperature treatment.

Among the transition metals, Zn^2+^ has hardly been considered as a dopant or codopant for ceria electrolytes, which may also be due to its expected limited solubility in the ceria lattice according to the Hume–Rothery rules. There are reports of using ZnO as a sintering aid since it lowers the sintering temperature as well as enhances the grain boundary conductivity in doped ceria.^24,25^ Ge et al.^24^ showed that a 1 mol % addition of ZnO to Ce_0.8_Ln_0.2_O_2−δ_ (Ln = Y, Sm, Gd) lowered the sintering temperature by ∼200 °C and enhanced the grain boundary conductivity.

This study attempts to investigate the effect of Bi^3+^ and Zn^2+^ as codopants for Ce_0.8_Sm_0.2–*x*_M*_x_*O_2−δ_ (where M = Bi^3+^ or Zn^2+^ and *x* = 0.05–0.2). The structure–property relationship, where the ionic conductivity is the property of interest for electrolytic applications, was studied for the codoped samples and compared to that of Ce_0.8_Sm_0.2_O_2−δ_. The anticipated role of carbonate in enhancing ionic conductivity was also considered.

## 2 Experimental Section

The solid solutions of Ce_0.8_Sm_0.2–*x*_M*_x_*O_2−δ_/carbonate (M = Bi^3+^ or Zn^2+^ where *x* = 0, 0.05, 0.1, 0.15, and 0.2) were prepared using the coprecipitation route. For Ce_0.8_Sm_0.2–*x*_Bi*_x_*O_2−δ_/carbonate where *x* = 0, 0.05, 0.1, 0.15, and 0.2, the sample IDs used are SBC0, SBC5, SBC10, SBC15, and SBC20, respectively. Similarly, SZC0, SZC5, SZC10, SZC15, and SZC20 were used for Ce_0.8_Sm_0.2–*x*_Zn*_x_*O_2−δ_/carbonate. The precursors Ce(NO_3_)_3_·6H_2_O, Sm(NO_3_)_3_·6H_2_O, and Bi(NO_3_)_3_·5H_2_O or Zn(NO_3_)_2_·6H_2_O (Sigma-Aldrich, 99.99% purity) were mixed according to their stoichiometric amounts in deionized water and stirred to dissolve all salts, followed by the dropwise addition of 2 mol L^–1^ of sodium carbonate solution (Sigma-Aldrich and 99.99% pure) to yield a total metal-ion-to-carbonate mole ratio of 1:2. The white precipitates obtained by vacuum filtration were dried in an oven at 120 °C and annealed at 400 °C for 4 h. The samples were then annealed at 800 °C, and part of the sample was further annealed at 1200 °C (for 4 h at each step). The obtained samples were ground into fine powders using a mortar and pestle for further characterization. A schematic of the synthetic method is shown in Figure S1.

## 3 Sample Characterization

The crystal structure was investigated using powder X-ray diffraction (XRD, Bruker D8, Germany) with Cu Kα radiation (λ = 1.54056 Å). Scanning electron microscopy (SEM, ThermoScientific Verios G4 UC or Regulus 8230, depending on availability) was used to record the micrographs of prepared samples. Raman spectrometry was also used to probe the structure of the powder samples (Renishaw inVia Reflex, λ = 532 nm). Fourier transform infrared spectroscopy (FTIR, ThermoFisher Scientific, Nicolet 6700) in the range from 4000 to 400 cm^–1^ was performed using the KBr disc method. The electrical properties of prepared pelletized samples were measured between 550 and 750 °C using electrochemical impedance spectroscopy (CorrTest Electrochemical using Corrosion Studio, Ver. 5.3, China) over a frequency range of 0.1 Hz–1 MHz and applying an amplitude of 10 mV. Circular pellets were prepared by pressing the powdered samples in an 11 mm diameter die at 250 MPa and then sintering in air for 4 h at 800 °C and 1200 °C depending on the annealing temperature. Pt paste was coated on both sides for current collection. Zview (Scribner) was used for equivalent circuit fitting.

### 3.1 Fuel Cell Measurement

Fuel cell performance (between 500 and 600 °C) was determined by a dc electronic load instrument (ITECH8511, ITECH Electrical Co., Ltd.). For this purpose, symmetrical button cells were prepared by sandwiching powders of selected samples (SBC5 and SZC5 annealed at 1200 °C) between commercially purchased Ni_0.8_Co_0.15_Al_0.05_LiO_2−δ_ (NCAL) electrodes (for both the anode and the cathode) and pressed in a 13 mm diameter die at 250 MPa. The button cells (∼1 mm thick with an active area of 0.64 cm^2^) were sintered at 600 °C for 1 h to dry and densify the Ag paste before testing. The performance of fuel cell was recorded at an elevated temperature using hydrogen as a fuel and air as an oxidant under laboratory conditions. Hydrogen (fuel, 99% pure) was fed at the anode with a flow rate of 120 mL/min, while air (oxidant) was supplied at the cathode with a flow rate of 100 mL/min.

## 4 Results and Discussion

### 4.1 Powder X-ray Diffraction (PXRD)

**Figure 1:**
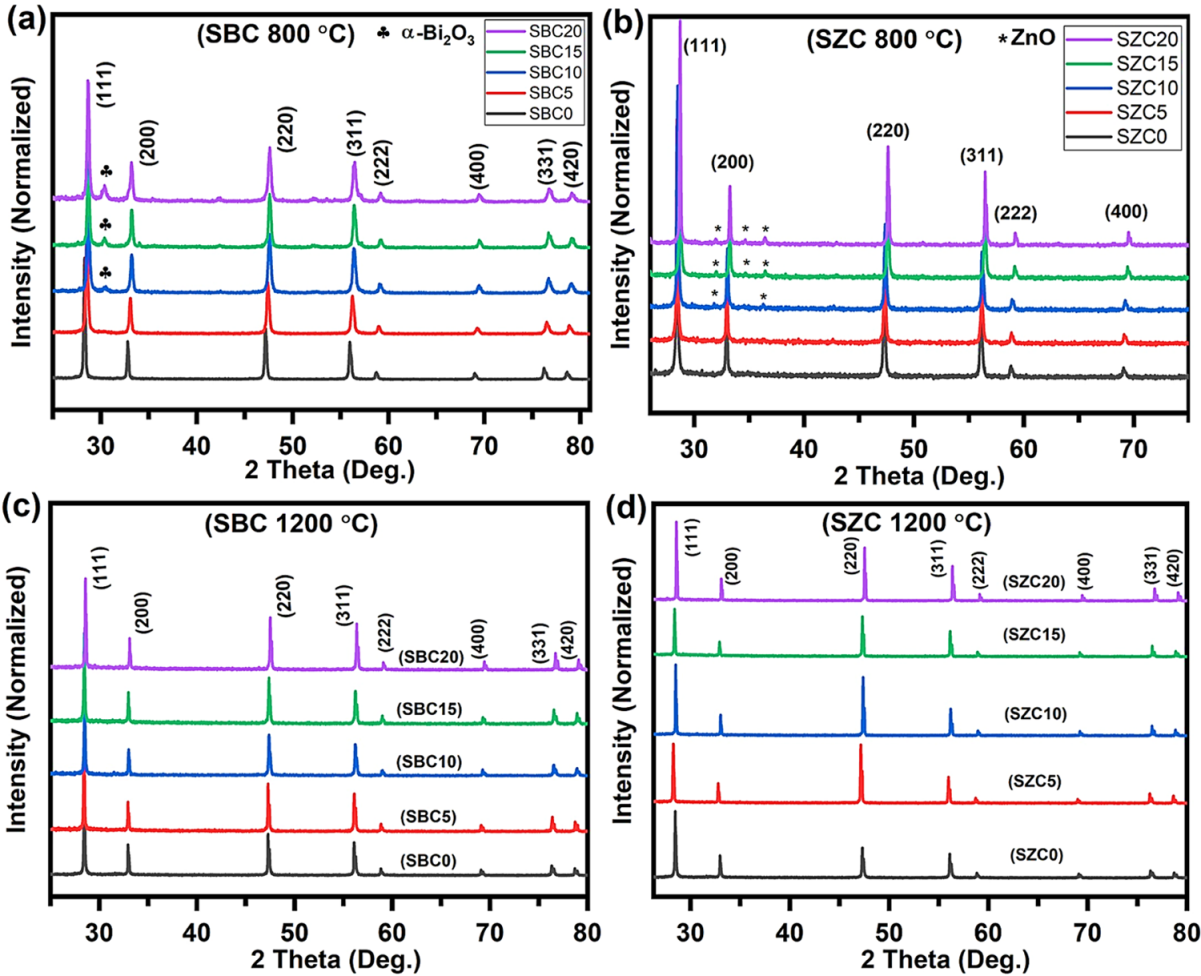
Room-temperature XRD patterns of samples annealed at 800 °C, showing the major fcc phase (indexed peaks) and the minor phases of (a) α-Bi_2_O_3_ in the SBC samples and (b) ZnO in the SZC samples as indicated. Upon further annealing at 1200 °C, the XRD patterns indicate a pure fcc phase for both (c) SBC and (d) SZC samples across the dopant range.

The room-temperature XRD diffraction patterns of SBC and SZC samples annealed at 800 °C are shown in Figure 1a,b. The diffraction patterns characteristic of face-centered cubic (fcc) ceria (space group *Fm*3̅*m*) are shown by all samples (ICSD # 00–034–0394).^26^ However, additional phases appear when larger amounts of Bi^3+^ or Zn^2+^ were added (above ∼10 mol %), which corresponded to a monoclinic α-Bi_2_O_3_ (space group *P*21/*c*; ICSD # 00–041–1449)^26^ for SBC and a hexagonal wurtzite ZnO (space group *P*63mc; ICSD # 00–036–1451)^26^ for SZC. This indicates that for the prepared samples that were annealed at 800 °C, the doping limit of the codopants (Bi^3+^ or Zn^2+^) into the Sm^3+^-doped ceria is ∼5 mol % (certainly less than 10 mol %) and the rest separates out to form a stable room-temperature α-Bi_2_O_3_ or ZnO phases. To assess the effect of higher-temperature annealing on the incorporation of codopants (Bi^3+^, Zn^2+^) into Sm^3+^-doped ceria, all samples were further annealed at 1200 °C and XRD patterns were recorded as depicted in Figure 1c,d. All samples now indicate the presence of only the fcc phase with no extra peaks. None of the XRD results picked up a carbonate phase, probably because this would be more amorphous.

The lattice parameters of the fcc phase were calculated using , and the results are presented in Table 1. As the ionic radius of Bi^3+^ (1.17 Å^27^) in an octahedral oxide arrangement is greater than that of Ce^4+^ (0.97 Å^27^) and Sm^3+^ (1.079 Å^27^), a slight systematic increase in the lattice parameter would have been expected as the amount of Bi^3+^ was increased with a corresponding decrease in Sm^3+^. Instead, a decrease in the lattice parameter was observed. For SBC samples annealed at 800 °C, Bi^3+^ was not fully incorporated in the ceria lattice due to the formation of the α-Bi_2_O_3_ phase and the decreasing Sm^3+^ content would justify the decrease in lattice parameter. Even for SBC5, the lattice parameter decreased, which may mean that not all of the added Bi^3+^ was incorporated in the lattice and that any additional phase was below the detection limit of XRD.

**Table 1:** Lattice Parameters (*a* in Å) for SBC and SZC Samples with Varied Dopant Concentrations That Were Annealed at 800 and 1200 °C, Respectively

sample	800 °C	1200 °C	sample	800 °C	1200 °C
SBC0	5.435	5.432	SZC0	5.435	5.432
SBC5	5.418	5.431	SZC5	5.427	5.447
SBC10	5.406	5.423	SZC10	5.419	5.421
SBC15	5.402	5.421	SZC15	5.400	5.427
SBC20	5.401	5.405	SZC20	5.391	5.403

When annealed at 1200 °C, the lattice parameters for SBC0 and SBC5 showed a very small difference indicating the incorporation of more Bi^3+^ into the ceria lattice. In fact for SBC5–SBC20, the lattice parameter increased when annealed at 1200 °C with respect to that for the samples annealed at 800 °C. This supports that the higher annealing temperature results in more successful doping of Bi^3+^ into the ceria lattice. SBC20 exhibited the smallest difference in the lattice parameter at the two annealing temperatures, which could indicate that a dissolution limit was reached and could also point to the role of Sm^3+^ in the lattice assisting in the incorporation of Bi^3+^ into the ceria lattice.

Different phases of Bi_2_O_3_ (e.g., α (monoclinic), β (tetragonal), γ (body-centered cubic), and δ (fcc)) are stable in different temperature ranges. α-Bi_2_O_3_ changes to δ-Bi_2_O_3_ at 730 °C,^28^ and this δ-phase can be stabilized to room temperature by doping.^29,30^ For the SBC samples annealed at 1200 °C, it is thus also possible that the small amount of the α-Bi_2_O_3_ phase was transformed into a δ-Bi_2_O_3_ phase and stabilized by the incorporation of Sm^3+^ or Ce^4+^ into the Bi_2_O_3_ lattice producing XRD peaks that would overlap with that of the fcc ceria phase. However, further characterization is required to verify this.

The SZC samples showed similar behavior to that of SBC. The ionic radius of Zn^2+^ (0.90 Å^27^) is smaller than that of Ce^4+^ and Sm^3+^; thus, an increasing amount of Zn^2+^ added (with a simultaneous decrease in Sm^3+^) would result in a decrease in the lattice constant. For samples annealed at 800 °C, the expected decrease in lattice parameter was observed. However, this clearly does not imply the successful incorporation of Zn^2+^ in the ceria lattice as the formation of a hexagonal wurtzite ZnO phase was observed to a small extent for SZC10–SZC20 and the decreasing Sm^3+^ content alone would cause a decrease in the cell parameter. Upon further annealing at 1200 °C, the hexagonal phase disappeared but the lattice parameters unexpectedly increased for all Zn^2+^-containing samples. Lin et al.^31^ also observed an expansion of the lattice parameter for 20 mol % of Zn-doped ceria upon increasing the annealing temperature (to 1300 °C). They suggested that Zn^2+^ could be incorporated into the ceria lattice either by substituting Ce^4+^ to create oxygen vacancies or by occupying interstitial sites causing the lattice to expand.

Lin et al.^31^ also noted that some Zn^2+^ crystallized out as ZnO for Zn-doped ceria calcined at 800 °C. After annealing at 1300 °C, a second-stage incorporation into the host ceria was observed, but using synchrotron-based XRD, they proved that the second-stage incorporation was still incomplete and the hexagonal ZnO phase was still present, although at much lower concentrations. It has also been shown that a cubic zinc-blende ZnO structure (space group *F*4̅3*m*) can be stabilized by growth on cubic substrates or with high-temperature and -pressure treatments.^32−34^ It is thus possible that as the SZC samples were annealed at 1200 °C, the wurtzite ZnO could also have transformed into a cubic-type ZnO where the XRD peaks would overlap with that for the doped ceria.

### 4.2 Raman Spectroscopy

**Figure 2:**
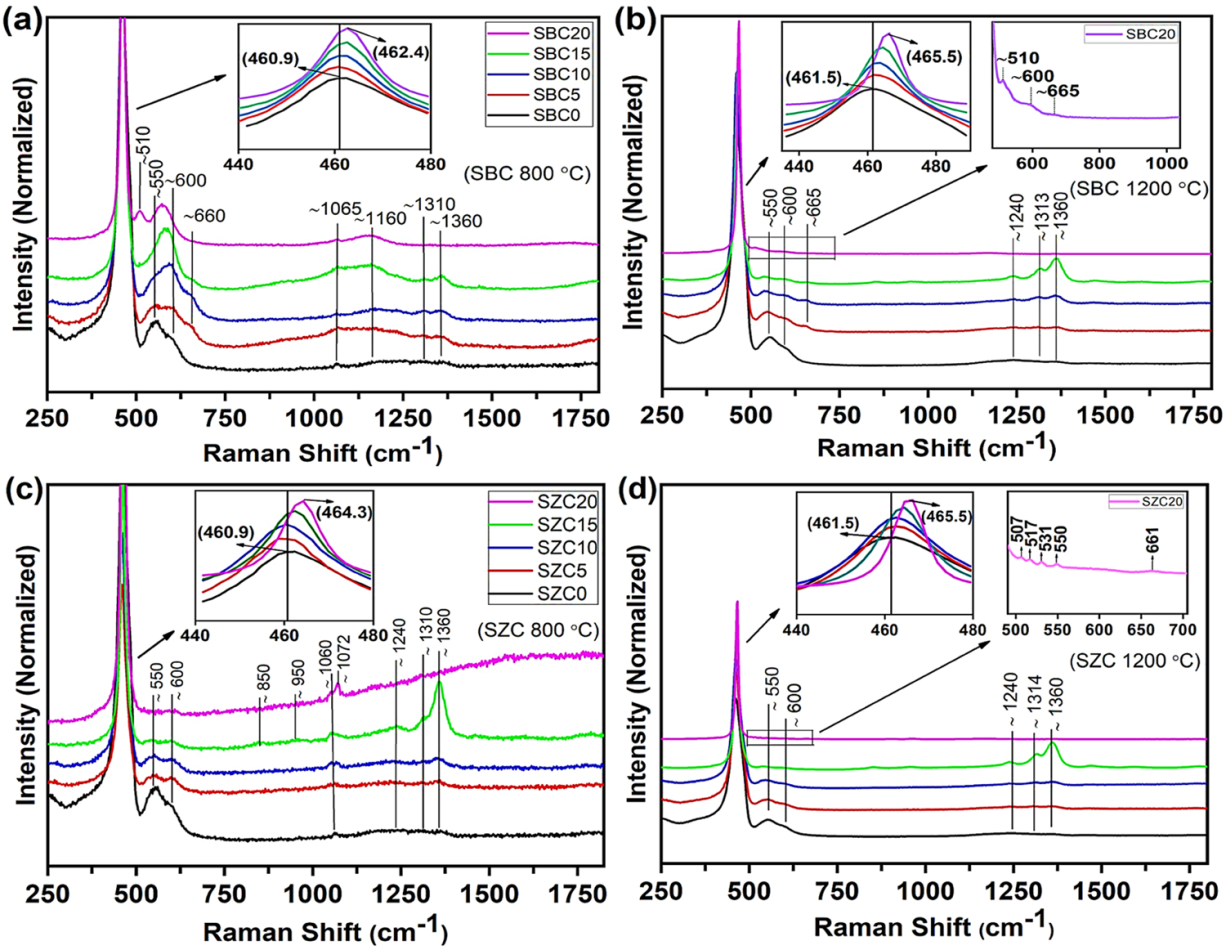
Raman spectra of SBC (a, b) and SZC (c, d) samples annealed at 800 and 1200 °C, respectively. The insets show enlarged regions of the spectra.

Figure 2a,b shows the room-temperature Raman spectra of SBC samples annealed at 800 and 1200 °C, respectively. For samples annealed at 800° C, the F_2g_ band can be seen to shift from 460.6 to 462.5 cm^–1^ (see Table 2) as the amount of Bi^3+^ added was increased together with a decrease in the amount of Sm^3+^ added. For pure ceria, the F_2g_ band was reported to occur at 462 cm^–1^ by Hebert and Stöwe^35^ and 465 cm^–1^ by Shirbhate et al.,^36^ noting that the morphology of the material can also affect the position of the band to a small extent.^37^ According to Sun et al.,^38^ ceria can compensate for high levels of oxygen vacancies due to the substitution of lower-valent elements in cation sublattice, leading to increased oxide ion conductivity. With the limited incorporation of Bi^3+^ in the ceria lattice, as evidenced by the presence of the α-Bi_2_O_3_ phase for SBC10–SBC20, it is expected that the F_2g_ band positions would be based mostly on the systematically decreasing Sm^3+^ content; hence, they shift towards the peak position for ceria. A similar trend was noted for the samples annealed at 1200 °C, with the F_2g_ bands occurring at a slightly higher wavenumber (see Table 2).

**Table 2:** F_2g_ Band Positions (in cm^–1^) with Varying Compositions for SBC and SZC Samples Annealed at 800 and 1200 °C, Respectively

sample	800 °C	1200 °C	sample	800 °C	1200 °C
SBC0	460.9	461.5	SZC0	460.9	461.5
SBC5	460.9	462.7	SZC5	460.9	462.5
SBC10	461.5	463.3	SZC10	460.9	462.8
SBC15	461.9	464.1	SZC15	462.6	464.3
SBC20	462.4	465.5	SZC20	464.3	465.5

For SBC0 (i.e., 20% Sm^3+^-doped ceria) annealed at 800 °C, the two peaks at ∼550 and ∼600 cm^–1^ represent the oxygen vacancies.^37^ With the increased Bi^3+^ addition and a coupled decreased Sm^3+^ content, the band at ∼550 cm^–1^ decreases in intensity, while the band at ∼600 cm^–1^ becomes broad and increases in intensity, and for SBC20, it shifts to a slightly lower wavenumber (∼570 cm^–1^). Another band at ∼510 cm^–1^ is also present for SBC20. Hebert and Stöwe^35^ also observed bands at 510 and 570 cm^–1^ for Ce_0.8_Bi_0.2_O*_x_* and Ce_0.9_Bi_0.1_O*_x_*, respectively, and assigned them to oxygen vacancy formation in ceria based on results from density functional theory (DFT) calculations by Schilling et al.^39^ A weak Raman peak ranging between 513 and 535 cm^–1^ for α-Bi_2_O_3_ has been reported,^28,40^ but other peaks at lower wavenumbers (e.g., 314 and 411 cm^–1^^35^) that are not overlapping with the ceria F_2g_ band were not present. For the samples annealed at 1200 °C, the peaks at ∼550 and ∼600 cm^–1^ remained in those positions for all samples with decreasing intensity as the ratio of Bi^3+^ to Sm^3+^ added increased, except for the SBC20 sample. On zooming in to this region for SBC20 (see the inset in Figure 2b), very low intensity peaks at ∼510 and ∼600 cm^–1^ were noted. No α-Bi_2_O_3_ was detected by XRD for samples annealed at 1200 °C. It thus suggests that the peak at ∼570 cm^–1^ for the 800 °C-annealed samples signifies that it is related to the α-Bi_2_O_3_ phase, which grows to become more dominant as the amount of Bi^3+^ is increased.

A broad band at ∼660 cm^–1^ for samples annealed at 800 °C and at ∼665 cm^–1^ for samples annealed at 1200 °C also appeared with the introduction of Bi^3+^, although not evident for SBC20. This can be ascribed to δ-Bi_2_O_3_ and which could not be detected using XRD due to the overlapping peaks with that for doped ceria. The broad band is due to a large amount of disorder in the structure and was observed by Vila et al. at around 610–640 cm^–1^,^28^ by Díaz-Guerra et al.^41^ at 630 cm^–1^ with a shoulder band at 655 cm^–1^ for a δ-Bi_2_O_3_ film, and by Rubbens et al.^42^ at ∼640 cm^–1^ for doped-Bi_2_O_3_. This band is not evident for SBC20 (and is very small for samples annealed at 1200 °C), which indicates that Sm^3+^ plays a role in stabilizing this δ-phase.

Deconvolution of the spectra up to 700 cm^–1^ was performed using a Gaussian amplitude function to determine more quantitative data (Figure S2). This was unfortunately not possible with the spectra for SBC5–SBC15 annealed at 800 °C due to the growing peak at ∼570 cm^–1^ overlapping the bands at both ∼550 and ∼600 cm^–1^. The defect concentration, determined as the area ratio between the ∼550 cm^–1^ and F_2g_ bands,^43^ the relative intensities of the oxygen vacancy band (shoulder band ∼550 cm^–1^), and the full width at half-maximum (FWHM) of the F_2g_ bands are compared schematically for the 1200 °C-annealed samples (Figure S3). The decreasing defect concentration with the systematically decreasing Sm^3+^ content highlights that the corresponding increase in the added codopant (Bi^3+^) does not result in the effective incorporation in the ceria lattice. The F_2g_ band becomes sharper and more intense from SBC0 to SBC20, supporting the hypothesis of the formation of more crystalline ceria phases due to minimal intrusion of impurities in the lattice.

The room-temperature Raman spectra of SZC samples annealed at 800 and 1200 °C are shown in Figure 2c,d, respectively. For SZC0–SZC10 annealed at 800 °C, the F_2g_ band is essentially at the same position (460.7 cm^–1^) (see the inset in Figure 2c and Table 2). For SZC15 and SZC20, the F_2g_ band position appears at higher wavenumbers (462.6 and 464.3 cm^–1^, respectively), possibly indicating a smaller extent of substitution into the ceria lattice, and this is supported by the XRD results showing the additional wurtzite ZnO. A similar trend was seen for the F_2g_ band positions for SZC samples annealed at 1200 °C (see Table 2).

The intensities of the oxygen vacancy bands at ∼550 and ∼600 cm^–1^ decrease with an increase in the Zn^2+^-to-Sm^3+^ ratio added, which again denotes lesser incorporation of Zn^2+^ into the ceria lattice. If Zn^2+^ were in the ceria lattice the intensity of the oxygen vacancy bands would have increased since fewer oxygens would be required to maintain charge neutrality. For SZC20, a minor band was seen at ∼600 cm^–1^ reflecting partial intrusion of Zn^2+^ to generate oxygen vacancies. Again, a similar trend was noted for samples annealed at 1200 °C. For SZC20, a closer look (see the inset in Figure 2d) reveals the presence of a few of very low intensity peaks at 507, 517, 531, and 550 cm^–1^ and a broad band at 661 cm^–1^, which are in reasonable agreement with those observed by Lima et al. for ZnO.^44^ These bands were not detected for SZC10 and SZC15, which was most likely due to the superimposition of these bands with the more pronounced shoulder bands in that region. Although not observed for SZC0–SZC15, SZC20 shows a slightly higher intensity peak at ∼1072 cm^–1^, which was ascribed to the presence of ZnO as noted according to the RRUFF Project.^45^

Deconvolution of the spectra for the SZC samples was performed as before (Figures S4 and S5), and the quantitative data was extracted (Table S2). The shoulder band at ∼550 cm^–1^ gradually weakened from SZC0 to SZC15, and for SZC20, a sharp F_2g_ band was present with no considerable shoulder band in the spectra of samples annealed at 800 and 1200 °C (Figure 2c,d). This clearly shows that a minimal amount of Zn^2+^ was incorporated in the ceria lattice (a separate ZnO phase was rather formed) and that only Sm^3+^ was substituted into the lattice resulting in the reduction of the defect concentration as the Sm^3+^ content was decreased. The defect concentration did not change significantly upon increasing the annealing temperature. The stepwise difference between the parameters (Table S2) upon changing the sample composition is not as large between SZC5 and SZC10 as compared to the others. This possibly signifies better intrusion of Zn^2+^ along with Sm^3+^ into ceria lattice for SZC10 compared to others.

The bands at ∼1240, ∼1310, and ∼1360 cm^–1^ in the spectra in Figure 2 are due to Sm^3+^ photoluminescence. These are clearly absent in the SBC20 and SZC20 samples where no Sm^3+^ is present. It is noted that the intensity of these bands increases with a decreasing Sm^3+^ content in all cases.

For samples annealed at 800 °C, there was evidence of some carbonate present. For the SBC samples, a characteristic band for carbonates in the ν_1_ region was located at ∼1065 cm^–1^^43^ (the broad band at ∼1160 cm^–1^ was attributed to the 2LO mode of ceria^46^). For the SBC samples, the peaks at ∼850 and ∼950 cm^–1^ were assigned to the presence of carbonate in the ν_2_ region.^47^ As these were all very low intensity peaks, the presence of carbonate was further investigated by FTIR. In all samples annealed at 1200 °C, these peaks disappeared, an expected outcome of high-temperature annealing.

### 4.3 FTIR Spectroscopy

**Figure 3:**
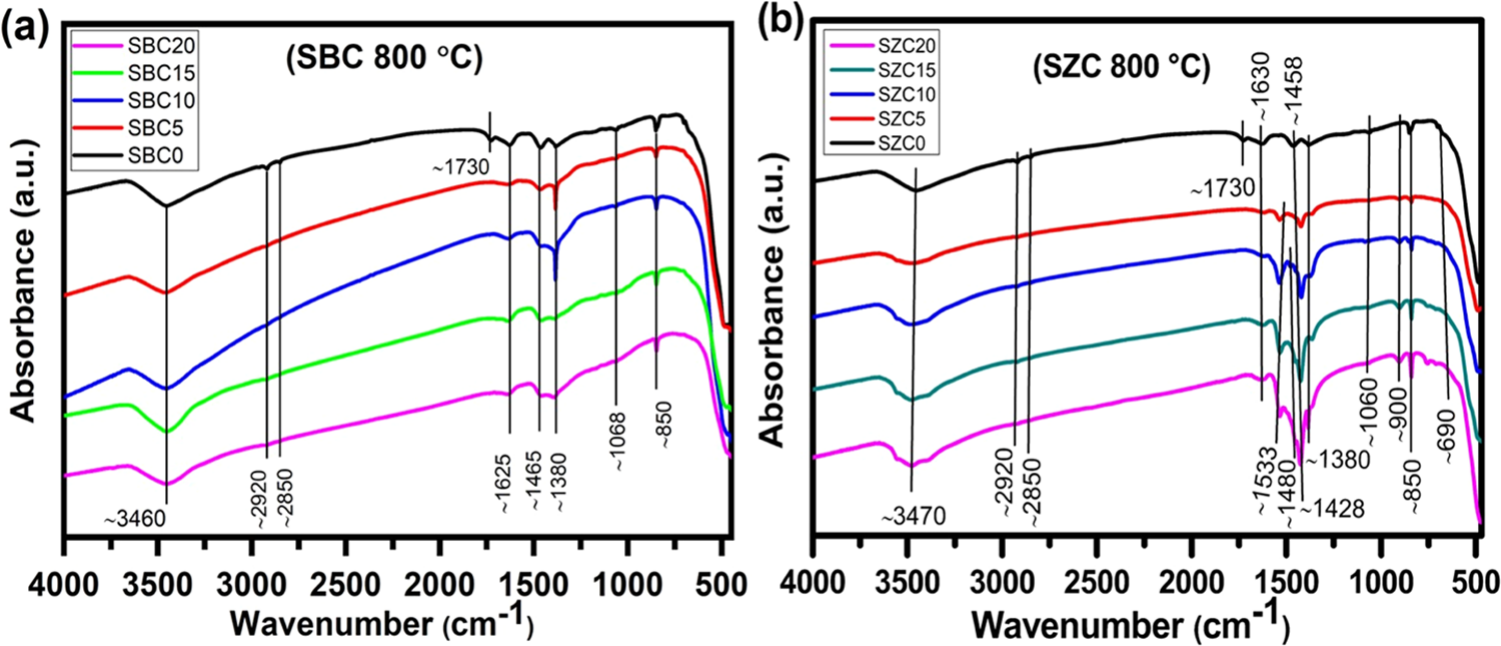
FTIR spectra of (a) SBC and (b) SZC samples annealed at 800 °C.

Figure 3 shows the FTIR spectra of the SBC (a) and SZC (b) samples annealed at 800 °C, respectively. The bands appearing at ∼3460, ∼2920, ∼2850, and 1625 cm^–1^ in (a) have been assigned to the O–H stretching, C–H stretching, and H–O–H bending, respectively.^48,49^ Another band only seen for SBC0 at 1731 cm^–1^ has been assigned to the presence of Na_2_CO_3_ on a ceria surface.^50,51^ Although not observed in Raman spectroscopy, FTIR also shows a band at ∼1465 cm^–1^, indicating the antisymmetric stretching of carbonate in the ν_3_ mode, similar to the 1444 cm^–1^ band for SDC/Na_2_CO_3_ reported by Yin et al.^51^ The presence of nitrates on the ceria surface was detected by the band located at ∼1380 cm^–1^.^46^ Raman spectroscopy indicated the presence of carbonate in the ν_1_ mode (symmetric stretching = 1000–1100 cm^–1^) at ∼1065 cm^–1^ and is observed at ∼1068 cm^–1^ in the FTIR spectra. Another band representing the out-of-plane bending of carbonate in the ν_2_ region (800–900 cm^–1^) was observed at ∼850 cm^–1^; however, it was not observed in Raman spectroscopy.^51^ The features below 700 cm^–1^ represent M–O vibrations.^50^ The FTIR spectra of SBC samples annealed at 1200 °C are represented in Figure S6a, where the bands associated with carbonates have completely vanished as previously noted due to the volatilization of the carbonates at this high temperature. The dominant bands observed are due to the physically adsorbed moisture (∼3460 and ∼1640 cm^–1^) and nitrates (∼1382 cm^–1^).^50−52^ As before, the M–O vibrations are present below 700 cm^–1^.^50^

The FTIR spectra of SZC samples (b) are similar to that of the SBC samples except for the ∼1533 and ∼1480 cm^–1^ bands, which appear to evolve and increase in intensity with increasing Zn^2+^ content. The former resembles the 1513 cm^–1^ band observed by Hu et al.^53^ who assigned it to polydentate carbonate on ZnO, while the latter, seen when the Zn^2+^ content is increased to 10 mol %, is in good agreement with the 1445 cm^–1^ band, indicating the presence of carbonate on a ZnO surface reported elsewhere.^54^ The FTIR spectra of SZC 1200 °C-annealed samples are like SBC samples (Figure S6b).

### 4.4 SEM

**Figure 4:**
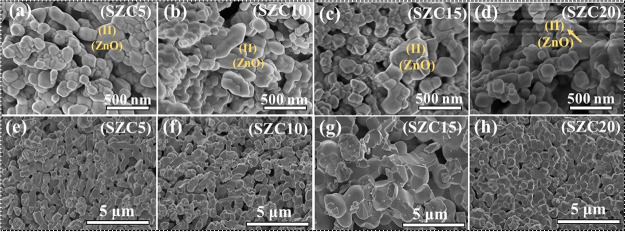
SEM micrographs of SZC5–SZC20 samples annealed at 800 °C (a–d) and 1200 °C (e–h), with (II) showing a possible ZnO-related phase.

Figure 4 depicts the microstructure of SZC samples annealed at 800 and 1200 °C, recorded using the ThermoScientific Verios G4 UC SEM. SZC0 comprised a range of shapes, with many being cubic along with some round and polygonal-shaped particles (see Figure S7a). For SZC5–SZC15 (800 °C annealed (a–d)), Zn^2+^ addition (with the corresponding Sm^3+^ removal) greatly affects the microstructure of ceria. The grain size distribution differs greatly with Zn^2+^ addition causing inhomogeneity. SZC20 shows particles of varying sizes. As XRD has detected the presence of ZnO for SZC10–SZC20, we may speculate that the unusual shaped particles may belong to ZnO, while the small round-shaped ones are those of ceria. Gao et al.^25^ studied the effect of ZnO on yttria-doped ceria and speculated that, after a certain solubility limit, excess ZnO tends to separate out in the form of larger zinc-rich particles or agglomerates.

The SEM micrographs of SZC samples annealed at 1200 °C (e–h) were taken using the Regulus SEM. The most obvious effect of high-temperature annealing is the particle growth. SZC0 comprises particles having polygonal and round morphologies (Figure S7b), while for SZC5, the introduction of Zn^2+^ affects the microstructure where particles tend to stick to each other leading to the formation of rod-shaped structures.

As the Zn^2+^ content is increased to 10 mol % (SZC10), small agglomerates are formed and do not display any particular morphology. With the further addition of the Zn^2+^ content up to 15 mol % (SZC15), the microstructure becomes relatively clear, with the majority being circular polygonal-shaped particles. For SZC20, particles of mixed morphologies can be seen, though the majority are polygonal.

Energy-dispersive system (EDS) analysis of samples SZC5–SZC20 annealed at 1200 °C was carried out and is represented in Figure S8. These indicate that even for the samples with the lowest Zn^2+^ content, i.e., SZC5, ZnO is observed. So, the solubility limit of Zn^2+^ in the ceria lattice is less than 5 mol %. For SZC10–SZC20, the separated ZnO-rich areas have clearly been shown by the EDS results in Figure S8.

**Figure 5:**
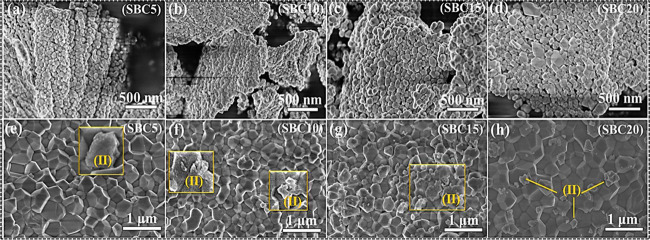
SEM micrographs of SBC5–SBC20 samples annealed at 800 °C (a–d) and 1200 °C (e–h). The insets labeled (II) in SBC5–SBC15 of 1200 °C-annealed samples are supposed impurity phases.

Figure 5a–d shows the surface morphologies of SBC5–SBC20 samples annealed at 800 °C and were recorded using the ThermoScientific Verios SEM. SBC0 is equivalent to SZC0 (both annealed at 800 and 1200 °C). For SBC5, the introduction of the Bi^3+^ content caused the formation of wire-like agglomerated structures. For SBC10–SBC15, particles of different sizes are visible with no distinctive morphology. The SBC20 micrograph depicts two different sized particles, which may represent two different phases, as indicated by both XRD and Raman spectroscopy.

The surface morphologies of the SBC samples annealed at 1200 °C were acquired using the Regulus 8230 SEM (Figure 5e–h). The high-temperature annealing again led to particle growth, evident from the SEM micrographs of all samples, and polygonal-shaped particles dominate the surface morphology. With the introduction of the Bi^3+^ content, some unusual particles begin to appear, which increase in quantity with increasing Bi^3+^ content. Although it is difficult to predict with certainty without EDS results, based on the results obtained by Raman spectroscopy and reasonably supported by XRD findings, we may speculate that these unusual particles may belong to the Bi_2_O_3_ impurity phase.

### 4.5 Electrochemical Impedance Spectroscopy (EIS)

**Figure 6:**
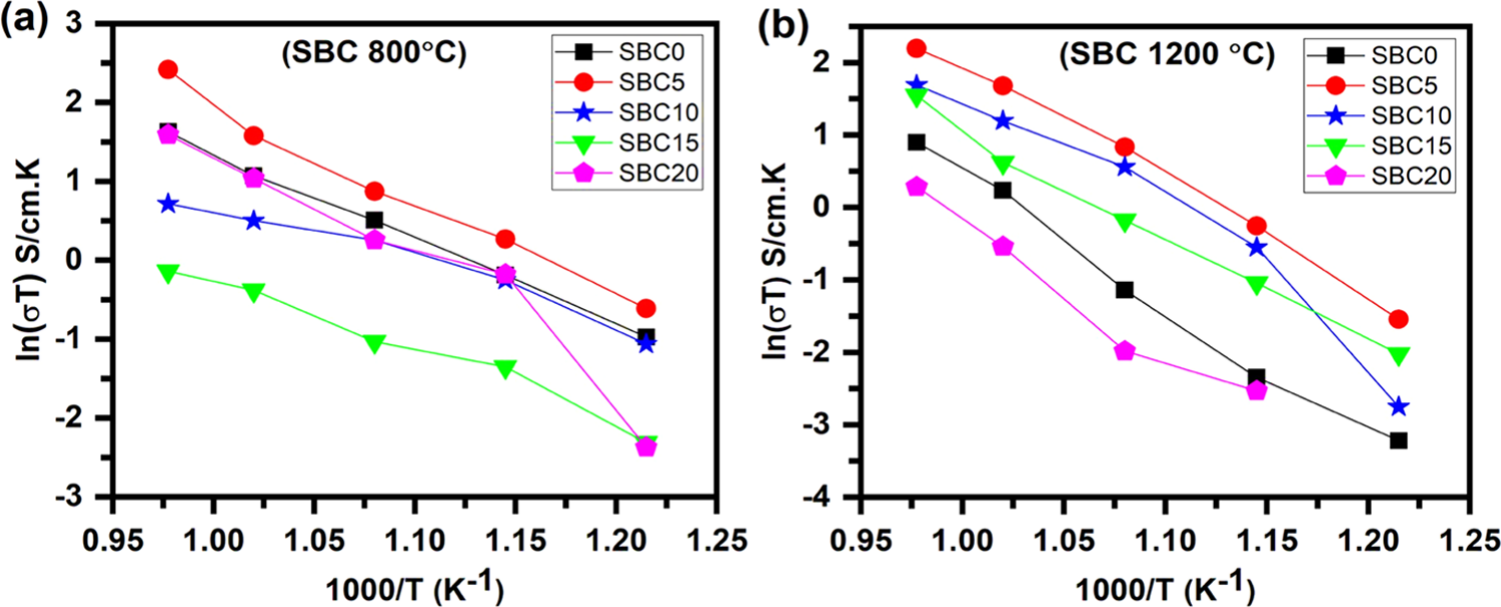
Arrhenius plots of SBC samples annealed at (a) 800 °C and (b) 1200 °C.

The electrochemical properties of SBC and SZC samples were measured over a temperature range of 550–750 °C in open air. The Nyquist plots of SBC samples annealed at 800 and 1200 °C are reproduced in Figure S9. For 800 °C-annealed samples in the frequency region used for measurements, SBC0 displayed predominantly the response due to the bulk and grain boundary, showing a decrease in resistance with an increase in the test temperature. At lower temperatures, the bulk region often did not completely resolve due to the high-frequency limitation of the instrument at 1 MHz. With the introduction of the Bi^3+^ content, particularly for SBC5–SBC15, predominantly the response due to the bulk and grain boundary region is observed. SBC15 shows a comparatively high resistance at low temperature. SBC20 shows a sharp decrease in resistance when the temperature is increased from 550 to 600 °C. This is evident in Figure 6a, where the Arrhenius plot of SBC20 indicates a stepped function in conductivity, where the reported conductivities incorporate both the bulk and grain boundary resistances.

As the presence of α-Bi_2_O_3_ was confirmed by XRD and Raman spectroscopy, the sudden increase in conductivity of SBC20 strongly suggests a phase change and the conductivity values recorded are most likely related to the more conductive δ-Bi_2_O_3_ phase. Interestingly, SBC10–SBC15 shows comparatively low conductivities among all samples (see Figure 6a). This may imply a combination of the ceria lattice having fewer oxide vacancies (due to the lower amount of Sm^3+^ added and Bi^3+^ not being significantly incorporated), as well as the secondary bismuth oxide phase not being present at a significant quantity to affect the conductivity. The conductivity was thus hampered by this α-Bi_2_O_3_ phase, which is not an ionic conductor.^55^ It is noted that the conductivity values at the lowest temperature are almost the same for SBC15 and SBC20. It appears that no phase change occurred in the case of SBC15 upon heating.

As XRD lattice parameters did not show the expected expansion and Raman spectra also indicated the presence of other Bi^3+^-related phases, the Nyquist plots of SBC samples annealed at 1200 °C will be affected by both the doped ceria phases and other Bi^3+^-related phases that are present (Figure S9). Compared to SBC0, there was a decrease in resistance for the 5 mol % doped Bi^3+^, i.e., SBC5. Comparable lattice parameter values for SBC0 and SBC5 mean the intrusion of Bi^3+^ into the ceria lattice to generate oxygen vacancies, which in turn means an increase in oxide ion conductivity. Interestingly, SBC10 and SBC15 indicate higher conductivity than SBC0, which might be due to the contribution from the Bi^3+^-related phase (δ-Bi_2_O_3_).

SBC20 showed a relatively different behavior with a significantly higher resistance at 550 °C being noted, so it has not been included. Even at 600 and 650 °C, it showed a comparatively higher resistance compared to SBC0. However, an abrupt change was noticed at 650 °C or above. A sudden drop in resistance is noticed, strongly suggesting the conduction due to the δ-Bi_2_O_3_-related phase, which is a high oxygen ion conductor. From Arrhenius plots in Figure 6b, the small step function of conductivity is particularly evident for SBC10 and SBC20, with SBC5–SBC15 showing higher conductivity values than SBC0 due to Bi^3+^addition, suggestive of ionic conduction due to the Bi^3+^-related phase.

In general, Figure 6 shows a decreasing trend in conductivities above 5 mol % Bi^3+^ content, particularly for 1200 °C-annealed samples, which might be due to nonincorporation of Bi^3+^ into ceria lattice and separation into a different phase, evident from the XRD lattice parameter trend and Raman spectroscopy results. The conductivities of 800 °C-annealed samples remain higher than those of 1200 °C-annealed samples pointing toward the possible contribution of carbonate in conductivity enhancement. Li et al.^56^ noted that the addition of Li_2_CO_3_ increases the grain boundary conductivity of SDC and lowers its sintering temperature. Different mechanisms for ion transport through the carbonate have been reviewed in detail in (17).

**Figure 7:**
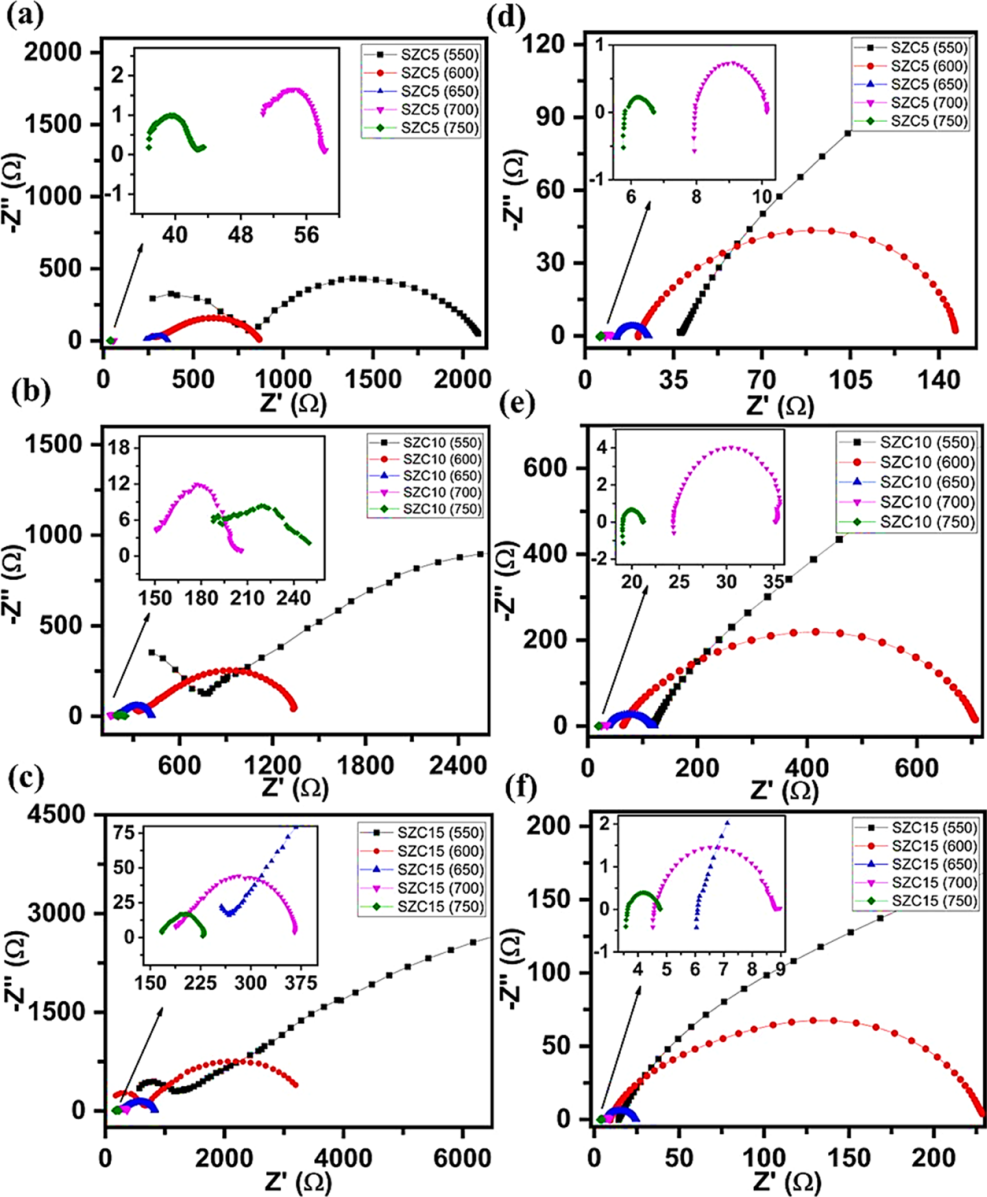
Nyquist plots of SZC5–SZC15 samples annealed at 800 °C (a–c) and 1200 °C (d–f), respectively.

Figure 7 shows the Nyquist plots of the SZC samples annealed at 800 and 1200 °C. SZC0 and SBC0 are the same samples (both 800 and 1200 °C annealed and see Figure S9). For the 800 °C-annealed samples, SZC5–SZC15 (Figure 7a–c) showed a noticeable bulk and grain boundary response with considerably higher resistances with increasing Zn^2+^ (and simultaneous decreasing Sm^3+^) content, which decreases significantly with increasing test temperature. The bulk response at lower temperatures could not be fully measured due to the limited test frequency. As XRD has indicated the presence of wurtzite ZnO and energy-dispersive X-ray (EDX) analysis also confirmed the Zn-rich regions, even for SZC5, it is believed that ZnO hinders the oxide ion conduction, thus decreasing the conductivity. SZC20 showed very high resistance, not suitable for electrolyte application and thus was not included here.

**Figure 8:**
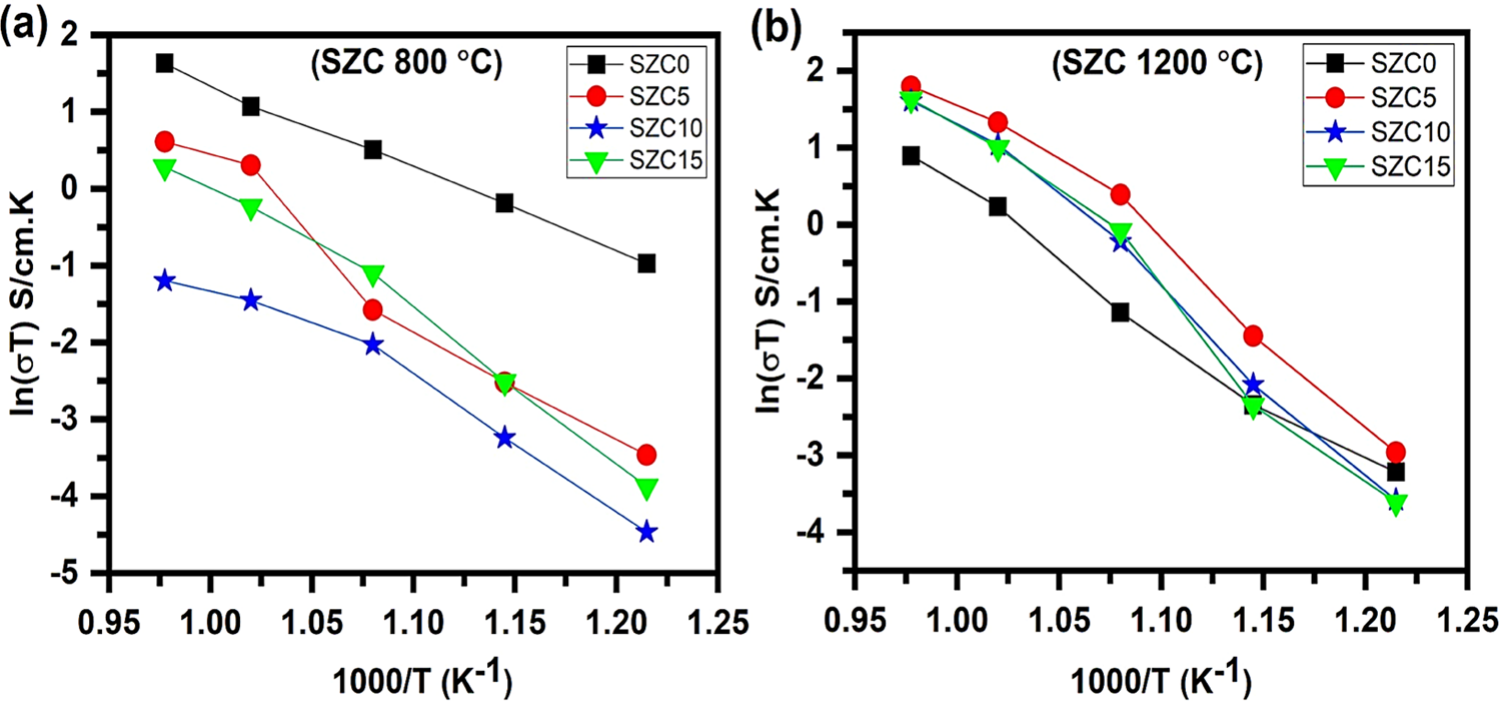
Arrhenius plots of SZC samples annealed at (a) 800 °C and (b) 1200 °C.

However, high-temperature annealing (1200 °C) introduces significant changes in Nyquist plots of SZC samples, depicted in Figure 7d–f. The charge-transfer resistance is considerably reduced for Zn^2+^-doped samples, particularly for SZC5, and with SZC10 and SZC15 being very similar (Figure 8b). The conductivities were now all higher than that of SZC0. The noticeable decrease in resistance might be due to the second-stage incorporation of Zn^2+^ in ceria to generate oxygen vacancies, as supported by XRD findings. It is interesting to note that the conductivity values follow a similar trend to the XRD lattice parameters of 1200 °C-annealed samples, i.e., increase in conductivity with increasing lattice parameter.

The Arrhenius plots of SZC samples annealed at 800 °C, shown in Figure 8a, indicate an overall decrease in conductivities with Zn^2+^ addition. As the presence of wurtzite ZnO was indicated for SZC5–SZC15, it appears that this phase, which is not an ionic conductor (rather it is a semiconductor^32^), together with the fact that the Sm^3+^ concentration is decreased in the ceria lattice, both lead to lower ionic conductivities compared to that for SZC0. SZC10 and SZC15 behaved similarly with activation energies of 1.56 and 1.77 eV, respectively, between 550 and 650 °C. The mechanism of conduction then changed as indicated by the decreasing slope and activation energies of 0.71 and 1.16 eV between 650 and 750 °C for SZC10 and SZC15, respectively. SZC5 showed an activation energy of 1.20 eV between 550 and 650 °C, with an abrupt change in conductivity when the temperature was increased from 650 to 750 °C, normally indicative of a phase change. However, variable temperature phase analysis would be required to fully confirm this.

The Arrhenius plots of SZC samples annealed at 1200 °C, depicted in Figure 8b, now show the Zn^2+^-doped samples having comparatively higher conductivity values than SZC0. SZC5–SZC15 gives steeper slopes when the temperature is increased from 600 to 650 °C, pointing toward the possible role of cubic-type ZnO in ionic conduction over 600 °C, particularly visible for Zn^2+^ content-containing samples.

The conductivities of Zn^2+^-doped samples annealed at 800 °C remain considerably lower compared to those annealed at 1200 °C (Table S5). SZC5 (1200 °C annealed) shows the best conductivity, with comparable values shown by SZC10 and SZC15, although reasonably higher than SZC0. The impedance data reveals that low-temperature annealing might be helpful to exploit the beneficial trait of carbonate in ionic conduction; however, it is not suitable for the effective incorporation of codopants into the host ceria lattice.

### 4.6 Fuel Cell Performance

**Figure 9:**
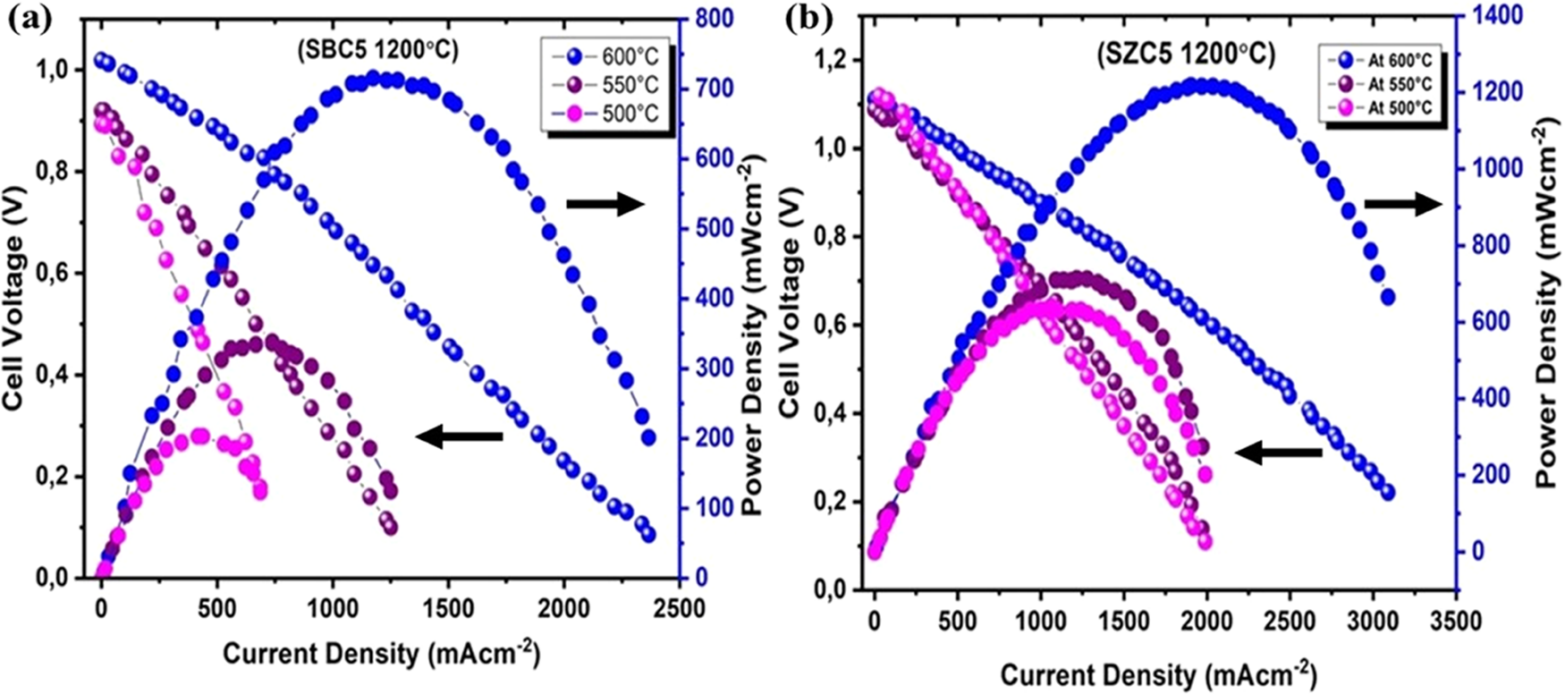
*I–V* and *I–P* curves of (a) SBC5 and (b) SZC5 both annealed at 1200 °C and measured from 500 to 600 °C.

The fuel cell performance of selected samples annealed at 1200 °C (SBC5 and SZC5) was recorded at a temperature range of 500–600 °C using hydrogen as a fuel and air as an oxidant under laboratory conditions. SBC5 showed slightly lower OCV at 500 °C, which increased with increasing test temperature, depicted in Figure 9, which might be due to the possible presence of α-Bi_2_O_3_ impeding the ionic conduction; however, it transforms into the δ phase at 600 °C, showing a sudden increase in OCV. The test temperature was confined to 600 °C. Above this temperature, Ni-foam shows stability issues, which may result in performance degradation. This is also sufficient as the aim is to look at the performance in the lower-temperature range (600 °C or lower). Figure 9 reflects a sharp increase in peak power densities for both samples when the test temperature was increased from 550 to 600 °C, strongly suggesting the performance improvement due to the presence of cubic-type phases of Bi_2_O_3_ and ZnO in SBC5 and SZC5, respectively. A remarkable increase in the peak power density (*P*_max_ = over 1230 mW/cm^2^) was recorded for SZC5, higher than SDC or other codoped samples reported in the literature, compared in Table S7.

## 5 Conclusions

Attempts were made to prepare codoped ceria powders via coprecipitation (using sodium carbonate) by systematically replacing Sm^3+^ with non-rare earth metals Bi^3+^ and Zn^2+^. For both dopant series annealed at 800 °C, a minimal incorporation of the codopants into host ceria occurred. This was observed by the decrease in lattice parameters and the F_2g_ band positions shifting to the pure ceria value with a decrease in defect concentration when Sm^3+^ was supposedly systematically replaced with Bi^3+^ or Zn^2+^. For 1200 °C-annealed samples, the incorporation of the codopants was slightly higher and additional δ-Bi_2_O_3_ and cubic-type ZnO phases appeared were present in the SBC and SZC powders, respectively.

EIS analysis of the SZ5–SZC15 codoped samples annealed at 800 °C showed lower conductivities compared to SZC0 (i.e., Ce_0.8_Sm_0.2_O_2−δ_). However, the same samples annealed at 1200 °C showed higher conductivity values than SZC0, suggesting that the cubic-type ZnO may assist in enhancing the ionic conductivity. SBC annealed at 800 and 1200 °C exhibited a similar trend. Exceptions were noted for the 800 °C-annealed SBC samples. SBC5 showed slightly higher conductivity than SBC0 (i.e., Ce_0.8_Sm_0.2_O_2−δ_) and SBC20 showed comparable conductivities to SBC0 when the test temperature was increased to 600 °C and above, which is believed to be due to the δ-Bi_2_O_3_ phase being fully formed as evident from the step function in the Arrhenius plot. Similar behavior was seen while measuring the fuel cell performance. For SBC5 and SZC5, the peak power densities recorded at 550 °C were close to 350 and 650 mW/cm^2^, respectively, which increased to ∼720 and ∼1230 mW/cm^2^ at 600 °C. This suggests that the performance improvement is due to the temperature-dependent phase change of the codopant oxide phases. This study suggests that the conductivity and performance of a solid electrolyte material can be tailored by the presence of multiple phases together with a ceria solid solution. However, the successful formation and effective control of these phases need further investigation in terms of thermal stability, mechanical stability (due to thermal expansion parameters), and the effect of aging on the conductivity properties.

## Supporting Information Available

Schematic of synthetics method and theoretical densities; multiple peak-fitted Raman spectra of SBC and SZC samples; quantitative data obtained by deconvolution of Raman spectra; FTIR spectra of SBC and SZC samples annealed at 1200 °C; SEM micrographs of SZC0; EDS images of SZC5–SZC20; Nyquist plots of SBC samples; total ionic conductivity values of SBC and SZC samples; error range of fitted resistances of SBC and SZC samples for EIS measurements; and a comparison of fuel cell performance data in a tabular form (PDF)
