# Supplementary material for: Phase-Assisted Tailored Conductivity of Doped Ceria Electrolytes to Boost SOFC Performance
Source: ACS Appl Mater Interfaces. 2023 Aug 9;15(33):39396–407. doi: 10.1021/acsami.3c08146 (PMC10450644; doi:10.1021/acsami.3c08146)
Supplement: Supplementary file 1 — am3c08146_si_001.pdf [file am3c08146_si_001.pdf]

Supporting Information

Phase assisted tailored conductivity of doped ceria electrolytes to boost SOFC performance.

Muhammad S. Arshad †,‡,§, Caren Billing †, David G. Billing †, Wanbing Guan §*

†Molecular Sciences Institute, School of Chemistry, University of the Witwatersrand, Private Bag X3, Johannesburg 2050, South Africa

‡Department of Chemical Sciences, University of Johannesburg, Doornfontein, Johannesburg 2028, South Africa

§Ningbo Institute of Material Technology and Engineering, Chinese Academy of Sciences, Ningbo, 315201, China

*Corresponding Author: Muhammad Sarfraz Arshad

E-mail address: 1669636@students.wits.ac.za

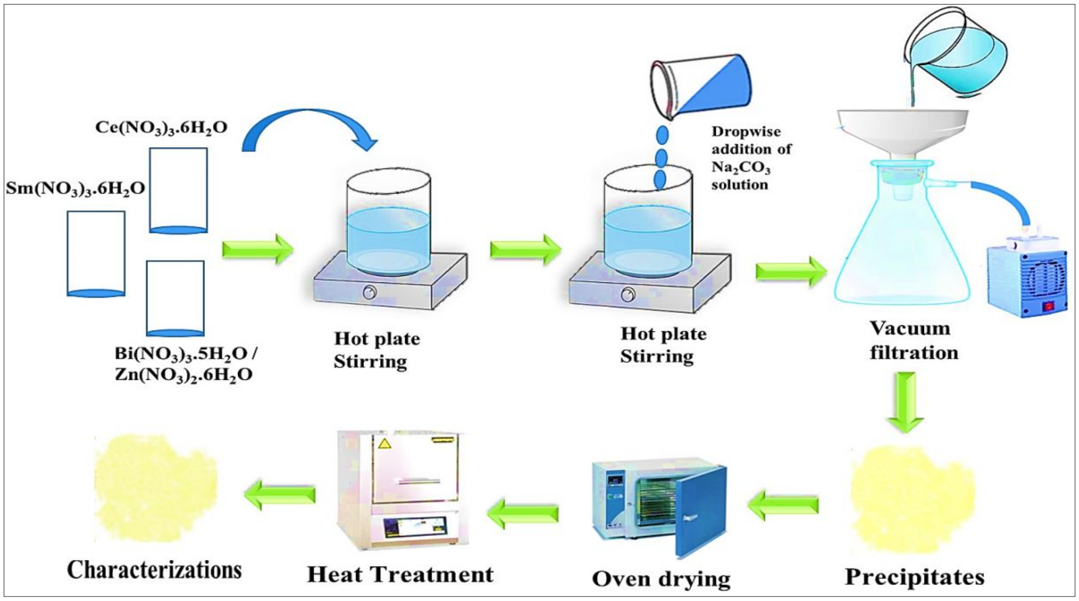

Figure S1. Schematic of the co-precipitation method for sample preparation.

Table S1. Theoretical densities (d_{th}: g/cm^3) of SBC and SZC samples annealed at 800 ˚C and 1200 ˚C, respectively.

Sample	800 ˚C	1200 ˚C	Sample	800 ˚C	1200 ˚C	Literature	d_{th} (g/cm^3)	Reference
SBC0	7.22	7.21	SZC0	7.22	7.21	CeO_2	7.26	[1]
SBC5	7.40	7.35	SZC5	7.06	6.99	SDC	7.16	[2]
SBC10	7.57	7.50	SZC10	6.92	6.91	$Ce_{0.8}Sm_{0.16}Zn_{0.04}O_{2-\delta}$	7.02	[3]
SBC15	7.72	7.62	SZC15	6.81	6.71	$Ce_{0.76}Gd_{0.}19Bi_{0.05}O1_{.88}$	7.49	[4]

SBC20	7.84	7.74	SZC20	6.67	6.62	$Ce_{0.85}Ca_{0.15}O_{2-x}$	6.96	[1]

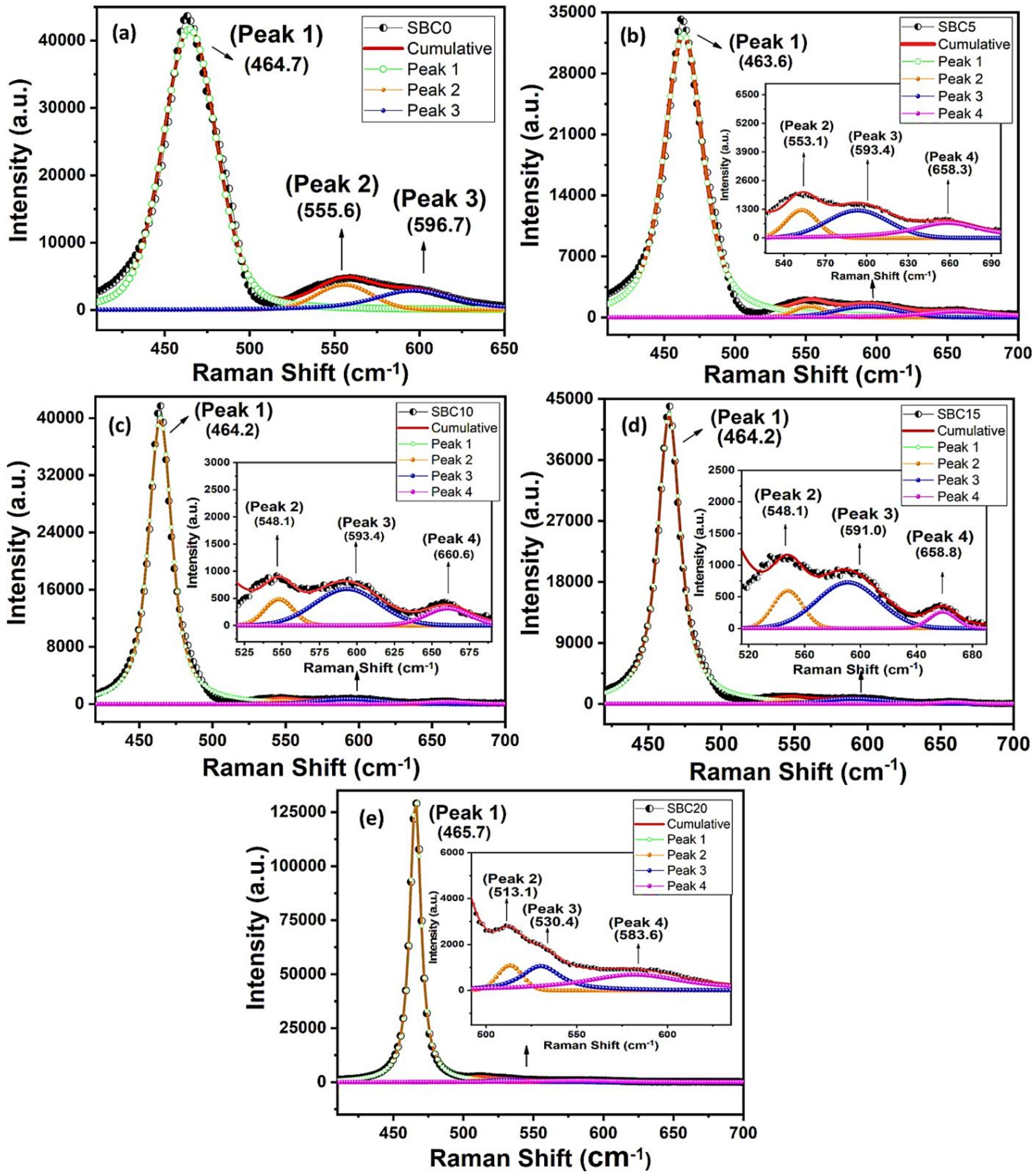

Figure S2. Multiple peak fitted Raman spectra of SBC samples annealed at 1200 ˚C (a) SBC0 (b) SBC5 (c) SBC10 (d) SBC15 (e) SBC20

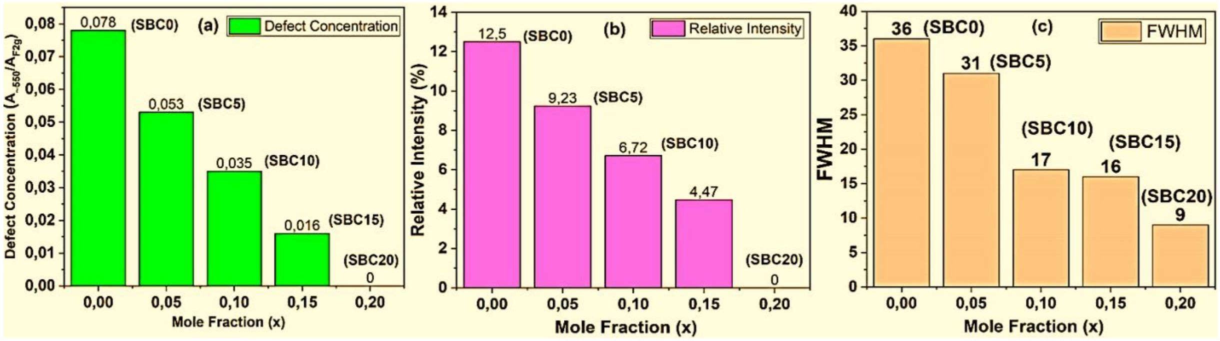

Figure S3. Estimated values derived from deconvolution of the Raman spectra for SBC samples annealed at 1200 ˚C showing (a) defect concentration, (b) relative intensities and (c) FWHM.

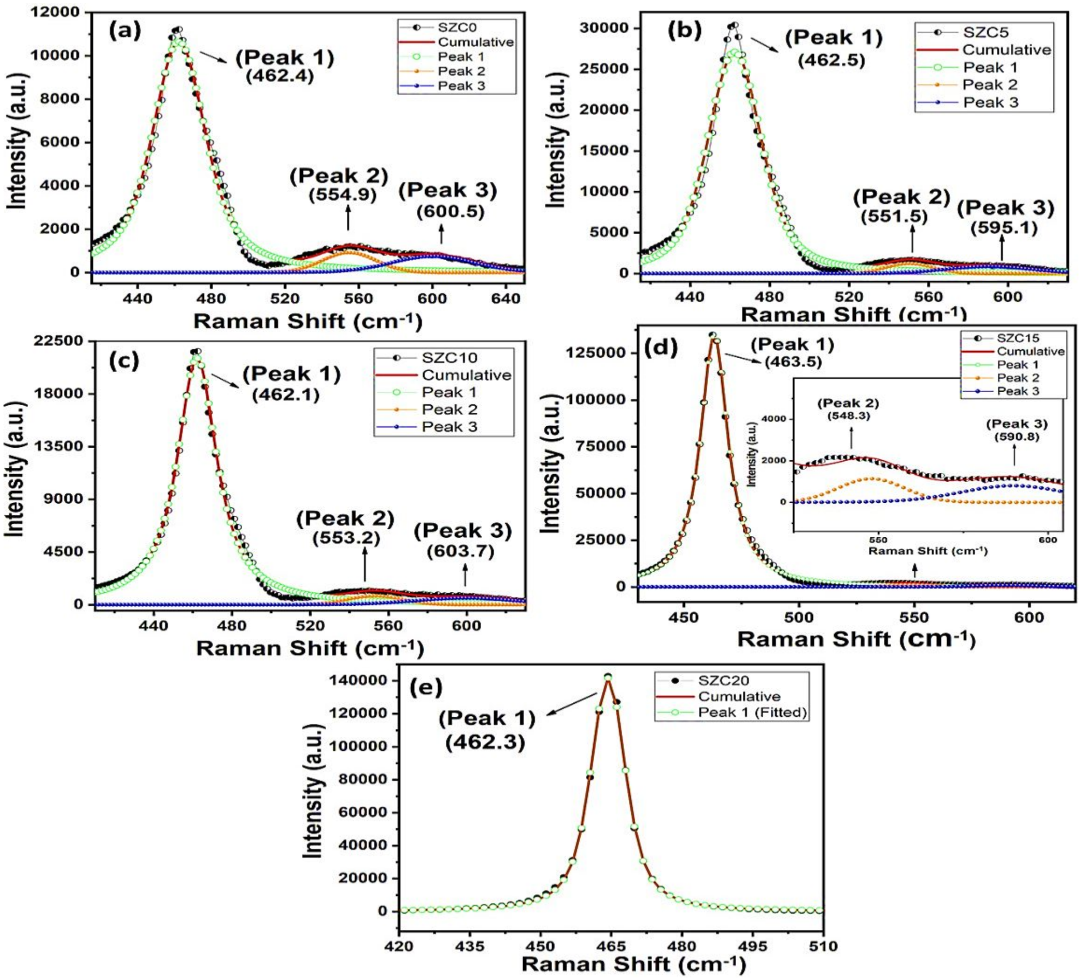

Figure S4. Multiple peak fitted Raman spectra of SZC samples annealed at 800 ˚C (a) SZC0 ((b) SZC5 (c) SZC10 (d) SZC15 (e) SZC20

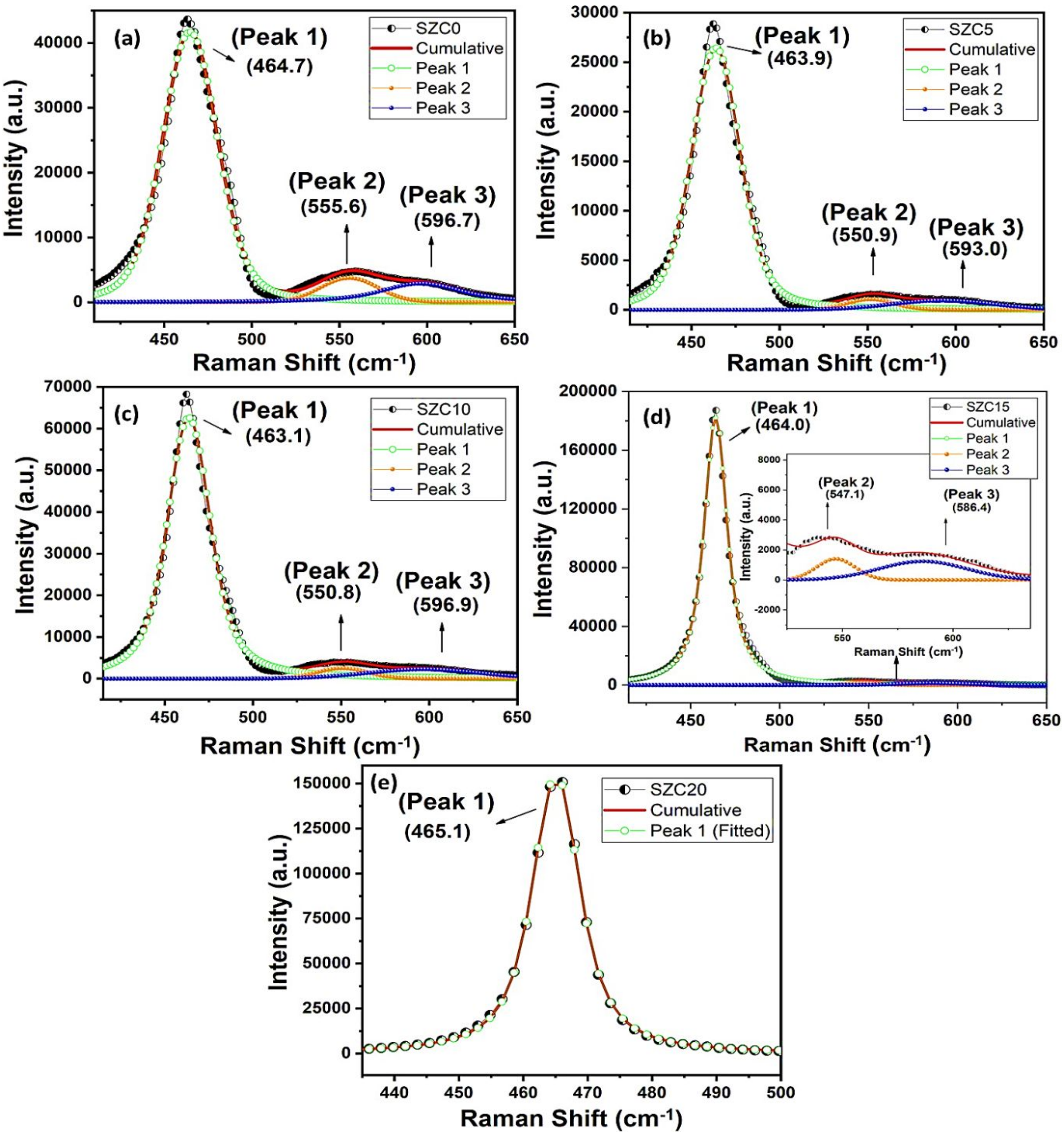

Figure S5. Raman spectra of SZC samples obtained after fitting and annealed at 1200 ˚C (a) SZC0 (b) SZC5 (c) SZC10 (d) SZC15 (e) SZC20

Table S2. Defect concentration, relative intensities and FWHM of SZC samples annealed at 800 ˚C and 1200 ˚C, respectively.

Sample	Defect Concentration ($A_{\sim550}/A_{F2g}$)		Relative Intensities* /%		FWHM /cm^{-1}	
	800˚C	1200˚C	800˚C	1200˚C	800˚C	1200˚C
SZC0	0.078	0.078	15.3	12.5	37.56	36.32
SZC5	0.045	0.047	11.8	10.6	29.05	30.75
SZC10	0.036	0.029	8.52	8.89	25.12	27.56
SZC15	0.013	0.014	4.72	4.79	14.67	14.79
SZC20	-	-	-	-	8.78	9.23

*Relative intensity – integrated area of ~550 cm^{-1} band

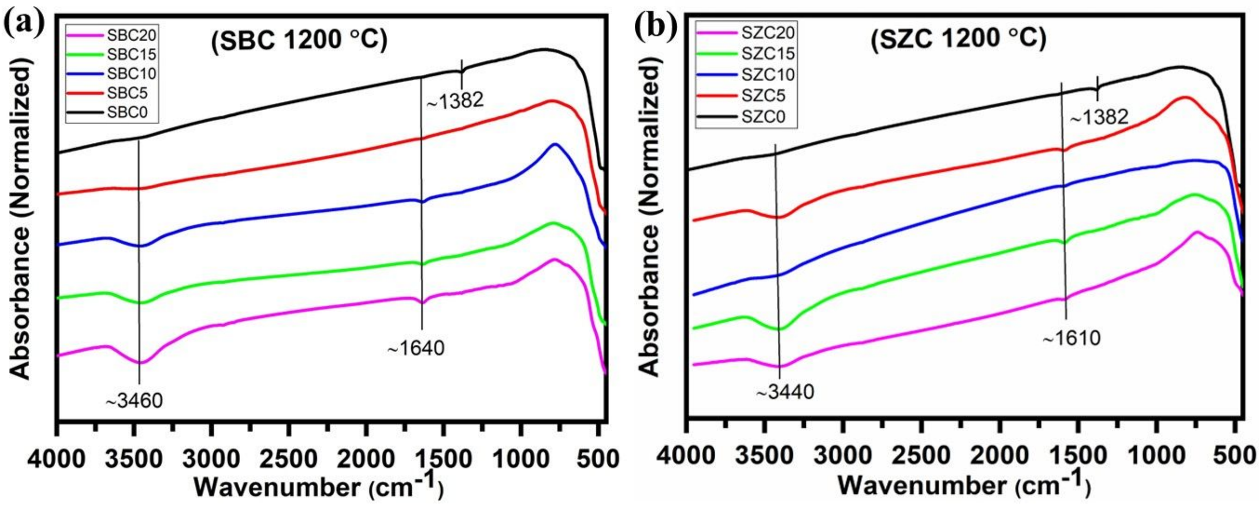

Figure S6. FTIR spectra of (a) SBC and (b) SZC samples annealed at 1200 ˚C, respectively.

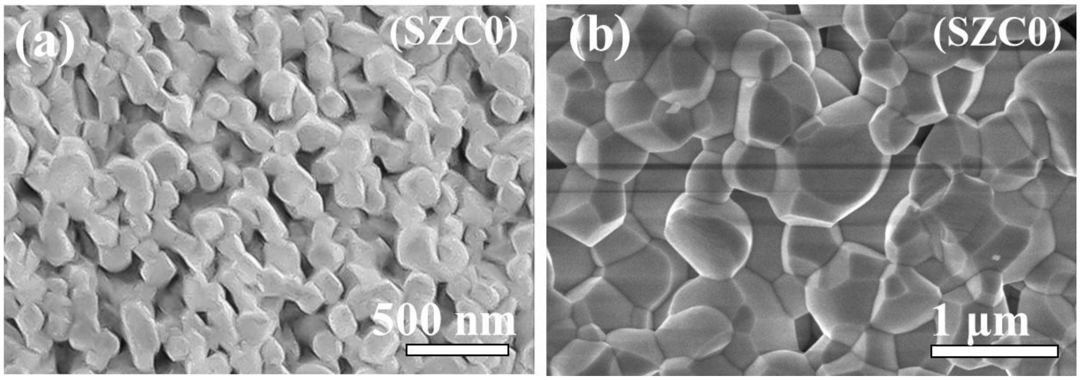

Figure S7. SEM micrographs of SZC0 annealed at (a) 800 ˚C and (b) 1200 ˚C, respectively.

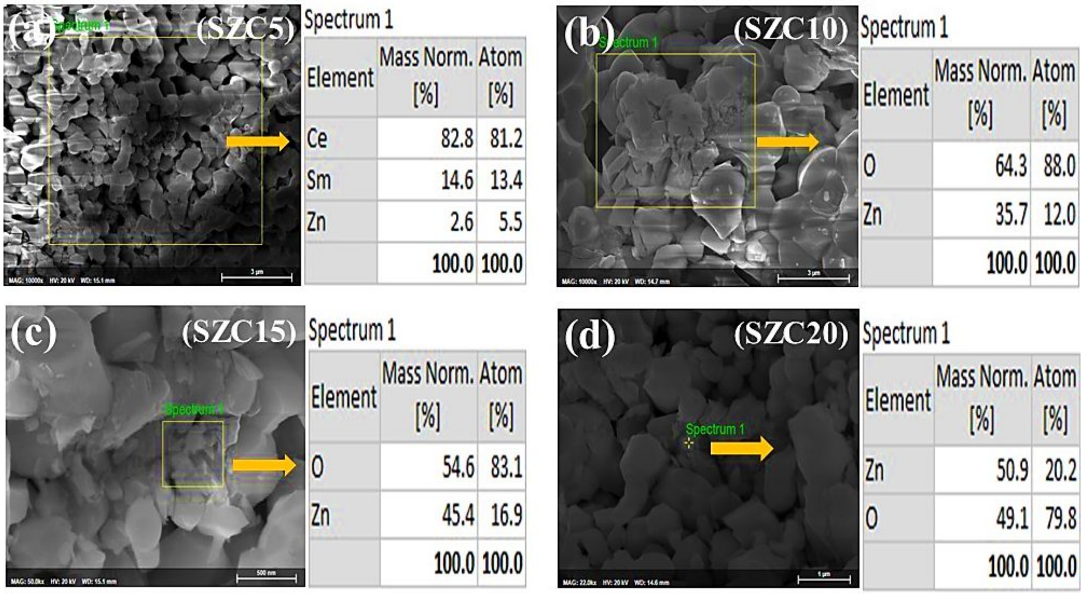

Figure S8. EDS images of SZC5 – SZC20 (a - d) annealed at 1200 ˚C and show accumulation of ZnO.

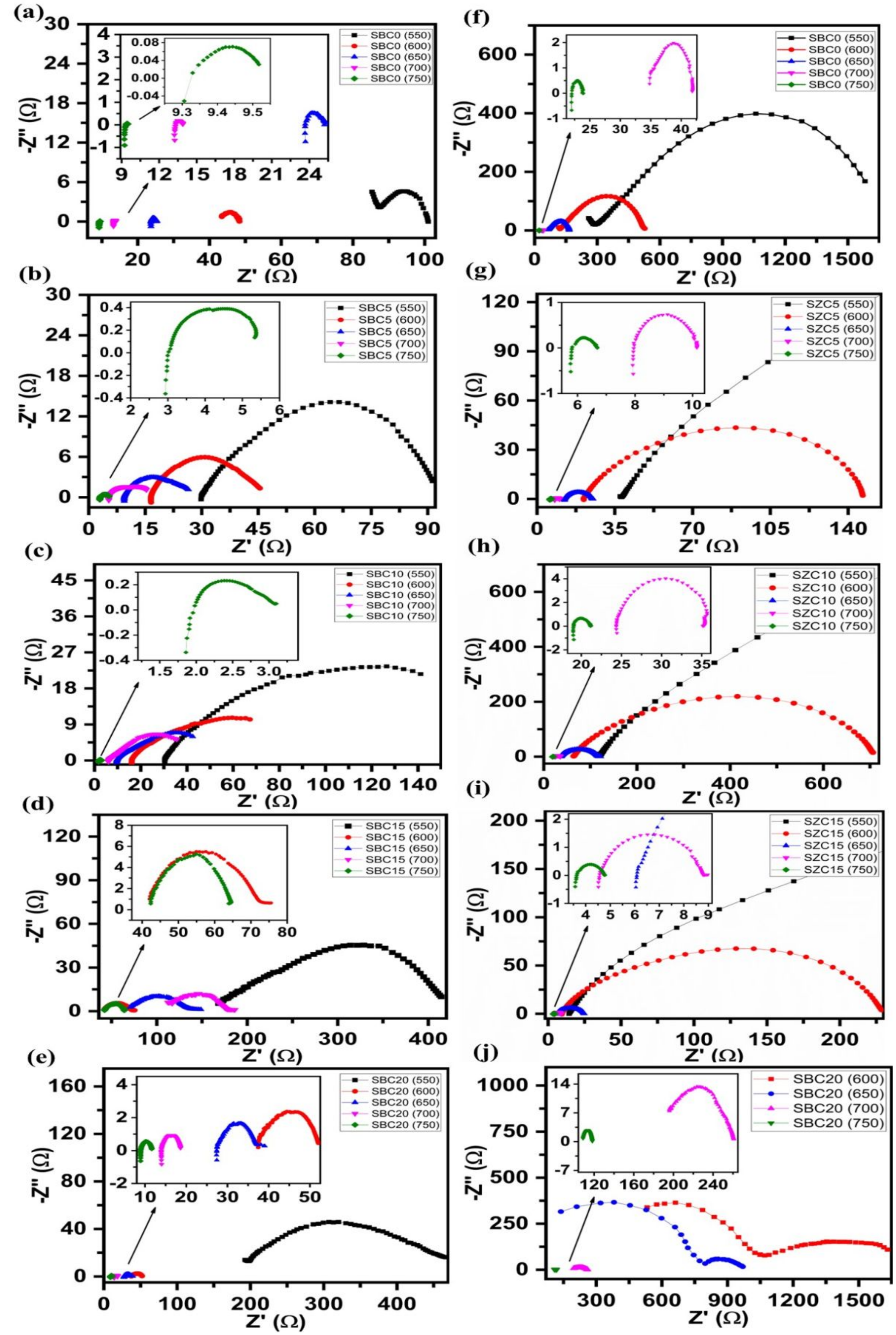
(a)
SBC0 (550)
SBC0 (600)
SBC0 (650)
SBC0 (700)
SBC0 (750)
-Z'' (Ω)
Z' (Ω)
(b)
SBC5 (550)
SBC5 (600)
SBC5 (650)
SBC5 (700)
SBC5 (750)
-Z'' (Ω)
Z' (Ω)
(c)
SBC10 (550)
SBC10 (600)
SBC10 (650)
SBC10 (700)
SBC10 (750)
-Z'' (Ω)
Z' (Ω)
(d)
SBC15 (550)
SBC15 (600)
SBC15 (650)
SBC15 (700)
SBC15 (750)
-Z'' (Ω)
Z' (Ω)
(e)
SBC20 (550)
SBC20 (600)
SBC20 (650)
SBC20 (700)
SBC20 (750)
-Z'' (Ω)
Z' (Ω)
(f)
SBC0 (550)
SBC0 (600)
SBC0 (650)
SBC0 (700)
SBC0 (750)
-Z'' (Ω)
Z' (Ω)
(g)
SZC5 (550)
SZC5 (600)
SZC5 (650)
SZC5 (700)
SZC5 (750)
-Z'' (Ω)
Z' (Ω)
(h)
SZC10 (550)
SZC10 (600)
SZC10 (650)
SZC10 (700)
SZC10 (750)
-Z'' (Ω)
Z' (Ω)
(i)
SZC15 (550)
SZC15 (600)
SZC15 (650)
SZC15 (700)
SZC15 (750)
-Z'' (Ω)
Z' (Ω)
(j)
SBC20 (600)
SBC20 (650)
SBC20 (700)
SBC20 (750)
-Z'' (Ω)
Z' (Ω)

Figure S9. Nyquist plots of SBC (SBC0 – SBC20) samples annealed at 800 ˚C (a – e) and 1200 ˚C (f – j), respectively.

In general, suitable equivalent circuits to fit the Nyquist plots were used depending on the sample and the measurement temperature and are based on the schemes given elsewhere [5]. Where the semi-circles were not fully measured, the resistance value was determined by extrapolating the curve and hence minor errors are introduced. These errors are not as obvious when taking the ln (natural logarithm) function of the conductivities to produce the Arrhenius plots.

Table S3. Temperature dependent conductivity values of SBC samples annealed at the two temperatures, displayed as σ_t(800 ˚C)/σ_t(1200 ˚C).

Total ionic conductivity, σ_t (S/cm) x 10^{-3}					
Sample	550˚C	600˚C	650˚C	700˚C	750˚C
SBC0	0.46/0.036	0.95/0.11	1.8/0.35	3.0/1.3	4.9/ 2.4
SBC5	0.66/0.26	1.5/ 0.89	2.6/ 2.5	5.0/ 5.5	11/8.8
SBC10	0.42/0.077	0.90/0.66	1.4/1.9	1.7/ 3.4	2.0/5.3
SBC15	0.12/ 0.16	0.30/0.41	0.39/ 0.91	0.70/1.9	0.85/4.6
SBC20	0.12/0.05	0.96/0.09	1.4/0.15	2.9/ 0.60	4.8/1.3

Table S4. Error range in % obtained by fitting the resistances of SBC samples across the temperature range 550 – 750 ˚C annealed at 800 ˚C and 1200 ˚C, respectively.

Error Range (%), 800 ˚C / 1200 ˚C					
Sample	550˚C	600˚C	650˚C	700˚C	750˚C
SBC0	0.16-1.04/ 0.96-1.68	0.14-1.14/ 0.73-1.16	0.26-5.19/ 0.56-0.85	0.25-6.58/ 0.17-1.08	0.19-13.7/ 0.14-1.89
SBC5	0.12-0.29/ 0.35-8.51	0.27-0.61/ 0.17-0.78	0.54-1.17/ 0.16-0.77	0.87-2.31/ 0.13-0.81	1.02-2.83/ 0.15-4.69
SBC10	0.25-0.93/ 0.29-2.16	0.57-1.79/ 0.47-1.06	1.19-3.54/ 0.29-0.86	0.97-4.62/ 0.28-1.18	0.77-1.80/ 0.33-3.34
SBC15	0.55-1.54/ 0.33-1.87	0.35-0.63/ 0.32-1.29	0.84-1.62/ 0.31-1.34	0.63-1.62/ 0.43-0.91	0.12-0.41/ 0.25-1.67
SBC20	3.34-6.87/ 0.54-0.80	1.37-2.91/ 0.14-0.60	1.16-3.91/ 0.15-0.85	0.28-0.86/ 0.08-0.41	0.45-2.14/ 0.10-0.53

Table S5. Temperature dependent conductivity values of SZC samples annealed at the two temperatures, displayed as σ_t(800 ˚C)/σ_t(1200 ˚C).

Total ionic conductivity, σt (S/cm) x 10^{-3}					
Sample	550˚C	600˚C	650˚C	700˚C	750˚C
SZC0	0.46/0.036	0.95/0.11	1.8/ 0.35	3.0/1.3	4.9/2.4
SZC5	0.038/0.063	0.092/0.27	0.22/1.6	1.4/ 3.9	1.8/ 5.9
SZC10	0.14/0.034	0.45/0.14	0.14/0.87	0.24/2.9	0.30/4.9
SZC15	0.025/0.033	0.094/0.11	0.36/1.0	0.82/2.8	1.3/ 5.0

Table S6. Error range (%) of fitted resistances for SZC samples annealed at 800 ˚C and 1200 ˚C across the temperature range 550 – 570 ˚C.

Error Range (%), 800 ˚C / 1200 ˚C					
Sample	550˚C	600˚C	650˚C	700˚C	750˚C
SZC0	0.16-1.04/ 0.96-1.68	0.14-1.14/ 0.73-1.16	0.26-5.19/ 0.56-0.85	0.25-6.58/ 0.17-1.08	0.19-13.7/ 0.14-1.89
SZC5	3.36-3.8/ 0.54-0.91	0.93-1.53/ 0.22-0.33	0.47-1.55/ 0.23-0.46	0.19-1.41/ 0.37-1.84	0.25-2.13/ 0.62-5.67

SZC10	1.39-2.29/ 0.57-0.66	3.64-3.97/ 0.32-0.35	0.46-1.11/ 0.21-0.23	0.26-1.20/ 0.23-0.78	0.75-3.39/ 0.16-1.58
SZC15	10.1-23.3/ 1.25-1.54	5.85-8.54/ 0.84-0.87	0.45-0.57/ 0.48-0.54	0.26-0.65/ 0.34-0.53	0.28-1.19/ 0.45-1.69

Table S7. A comparison of peak power densities (mW/cm^2) of lab scaled SOFCs with ceria based electrolytes measured at different temperatures.

Electrolyte	Temperature (˚C)	Peak Power Density	Reference
$Sm_{0.2}Ce_{0.8}O_2$-Na_2CO_3	600	281.5	[6]
$Sm_{0.2}Ce_{0.8}O_{1.9}$-Na_2CO_3	550	421	[7]
$Sm_{0.2}Ce_{0.8}O_2$-$BaCO_3$	600	427	[8]
$Sm_{0.2}Ce_{0.8}O_{1.9}$	700	366	[9]
$Ce_{0.8}Y_{0.2}O_{2-\delta}$	800	310	[10]
$Ce_{0.8}Y_{0.15}Ca_{0.05}O_{2-\delta}$	800	510	[10]
$Ce_{0.8}Sm_{0.15}Bi_{0.05}O_{2-\delta}$	600	720	This study
$Ce_{0.8}Sm_{0.15}Zn_{0.05}O_{2-\delta}$	600	1239	This study

References

[1] Parkash, O.; Singh, N.; Singh, N. K.; Kumar, D. Preparation and Characterization of Ceria Co-Doped with Ca and Mg. Solid State Ion. 2012, 212, 100–105.

[2] Ahmad, S. I.; Mohammed, T.; Bahafi, A.; Suresh, M. B. Effect of Mg Doping and Sintering Temperature on Structural and Morphological Properties of Samarium-Doped Ceria for IT-SOFC Electrolyte. Appl. Nanosci. 2017, 7 (5), 243–252.

[3] Yang, T.; H, Qian.; Y, Fang.; Y, Fang.; F, Hao.; Z, Chunhua. Low Temperature Densification and Electrochemical Properties of Sm0.16Ce0.84O1.92 Electrolyte by Zn Doping. Int. J. Electrochem. Sci. 2016, 5724–5734.

[4] Accardo, G.; Frattini, D.; Ham, H. C.; Han, J. H.; Yoon, S. P. Improved Microstructure and Sintering Temperature of Bismuth Nano-Doped GDC Powders Synthesized by Direct Sol-Gel Combustion. Ceram. Int. 2018, 44 (4), 3800–3809.

[5] Xia, Z.; Meng, B.; Zhang, H.; Zheng, Q.; Liang, W.; Ping, X. Effects of MgO Additions on the Electrical Conduction Behavior of a CeO2-Based Electrolyte Prepared by SPS Process Ceram. Int. 2020, 46, 9622–9628.

[6] Zhang, G.; Li, W.; Huang, W.; Cao, Z.; Shao, K.; Li, F.; Tang, C.; Li, C.; He, C.; Zhang, Q.; Fan, L. Strongly Coupled Sm0.2Ce0.8O2-Na2CO3 Nanocomposite for Low Temperature Solid Oxide Fuel Cells: One-Step Synthesis and Super Interfacial Proton Conduction. J. Power Sources 2018, 386, 56–65.

[7] Li, C.; Zeng, Y.; Wang, Z.; Ye, Z.; Zhang, Y. Processing Temperature Tuned Interfacial Microstructure and Protonic and Oxide Ionic Conductivities of Well-Sintered Sm0.2Ce0.8O1.9-Na2CO3 Nanocomposite Electrolytes for Intermediate Temperature Solid Oxide Fuel Cells. J. Power Sources 2017, 360, 114–123.

[8] Khan, A.; Wang, Y.-H.; Hung, I.-M. Characterization and Performance of a Samarium-Doped Ceria/Barium Carbonate Composite Electrolyte for Low-Temperature Solid Oxide Fuel Cells. J. Electron. Mater. 2022, 51 (3), 1013–1020.

[9] Cheng, J.; Qian, W.; Wang, P.; Tian, C. A High Activity Cathode of Sm0.2Ce0.8O1.9 Decorated Mn1.5Co1.5O4 Using Ion Impregnation Technique within a Solid Oxide Fuel Cell System. Solid State Sci. 2022, 131 (106962), 106962.

[10] Kang, J.; Feng, W.; Guo, D.; Chen, K.; Gao, S.; Jiang, J.; Lu, C.; Niu, B.; Wang, B.

Performance Optimization of Ca and Y Co-Doped CeO2-Based Electrolyte for Intermediate-Temperature Solid Oxide Fuel Cells. J. Alloys Compd. 2022, 913 (165317), 165317.
